# High-Risk Factors of In-Hospital Death Following Complex High-risk and Indicated Patients After Percutaneous Coronary Intervention Supported by Extracorporeal Membrane Oxygenation

**DOI:** 10.31083/RCM27126

**Published:** 2025-05-26

**Authors:** Wenjie Qiu, Wanying Chen, Yajun Qin, Yifang Zhou, Yuanshen Zhou

**Affiliations:** ^1^The Second Clinical College of Guangzhou University of Chinese Medicine, 510006 Guangzhou, Guangdong, China; ^2^Department of Cardiovascular, Guangdong Provincial Hospital of Chinese Medicine, 510120 Guangzhou, Guangdong, China

**Keywords:** complex high-risk and indicated patients, percutaneous coronary intervention, extracorporeal membrane oxygenation, risk factors of in-hospital death, meta-analysis

## Abstract

**Background:**

Complex high-risk and indicated patients (CHIPs) increase the risk of in-hospital death after percutaneous coronary intervention (PCI). Extracorporeal membrane oxygenation (ECMO) support can improve survival. However, there remains a gap in knowledge regarding how to identify and manage these high-risk patients effectively to reduce mortality. This study aimed to determine the independent high-risk factors associated with increased risk of in-hospital mortality among CHIPs after PCI with ECMO support. This research focused on providing clinicians with more accurate risk assessment tools for devising more effective treatment plans for these patients.

**Methods:**

The EMBASE, PubMed, Cochrane Library, Web Of Science, Chinese Biomedical Database, China National Knowledge Infrastructure, China Science and Technology Journal Database, and Wanfang databases were searched from their inception to October 1, 2024, to identify observational studies examining mortality risk amongst adult CHIPs (age ≥18 years). The primary outcome was in-hospital mortality. A meta-analysis used random-effects models to obtain summary odds ratios (ORs) with 95% confidence intervals (CIs). The Cochrane risk-of-bias tool assessed the quality of evidence.

**Results:**

Ten studies with 306 participants were included. In pooled analyses, cardiogenic shock (CS) or cardiac arrest (CA) to ECMO (mean difference (MD) : 34.61, 95% confidence interval (CI): 26.70 to 42.52; *p *< 0.00001), ECMO duration (MD : –19.93, 95% CI: –32.85 to –7.02; *p* = 0.002), type of infarction-associated coronary artery-left anterior descending (LAD; OR : 3.16, 95% CI: 1.83 to 5.47; *p* < 0.0001), body mass index (BMI; MD: 1.52, 95% CI: 1.06 to 1.97; *p* < 0.00001), lactate levels (MD: 3.15, 95% CI: 2.37 to 3.94; *p* < 0.00001), left ventricle ejection fraction (LVEF; MD: –4.09, 95% CI: –6.17 to –2.00; *p* = 0.0001), mean arterial pressure (MAP; MD: –24.92, 95% CI: –32.19 to –17.65; *p* < 0.00001), heart rate, male sex, left circumflex, and right coronary artery, were associated with in-hospital mortality.

**Conclusions:**

CHIPs with longer CS or CA to ECMO, shorter ECMO duration, LAD infarction, higher BMI, elevated lactate levels, and lower LVEF and MAP have an increased risk of in-hospital death.

## 1. Introduction

With changes in lifestyle and increased life expectancy, the prevalence of patients characterized as complex high-risk and indicated patients (CHIPs) is on the rise, coinciding with an increased incidence of acute myocardial infarction (AMI) in these patients [1]. CHIPs refers to patients with complex, high-risk, and intervention-indicated conditions, including those who are at significant risk or have contraindications for surgical treatment [2]. Percutaneous coronary intervention (PCI) may be their only chance of survival. Nevertheless, these patients often have complex coronary lesions, numerous clinical comorbidities, and poor cardiac function. Accordingly, the probability of complications, including ischemia, heart failure, malignant arrhythmia, and lack of blood flow during PCI increases, and the patient’s ability to tolerate myocardial ischemia caused these complications decreases [3].

CHIPs face high in-hospital mortality and poor prognosis due to their complex conditions and high risk of disease. Faced with the increased risk of hemodynamic collapse and death, patients can be supported with extracorporeal membrane oxygenation (ECMO), which aids pulmonary and cardiac functions via veno-venous and veno-arterial configurations [4]. ECMO, a combined blood pump and oxygenator, is widely used to assist in the treatment of CHIPs after PCI, and significantly reduces the mortality of CHIPs. However, no systematic studies have evaluated the impact of ECMO support on post-PCI mortality in CHIPs [5]. Therefore, the current study seeks to explore the risk factors of in-hospital death after PCI in CHIPs requiring ECMO adjuvant therapy.

While there have been several systematic reviews and research on CHIPs and ECMO support, none have specifically focused on the impact of ECMO support on post-PCI mortality in this patient population. This study aims to fill that gap by identifying the independent risk factors associated with increased risk of in-hospital mortality among CHIPs after PCI with ECMO support. Through this research, we aim to provide clinicians with more accurate risk assessment tools for devising more effective treatment plans for these patients.

## 2. Methods

**Fig. 1.**
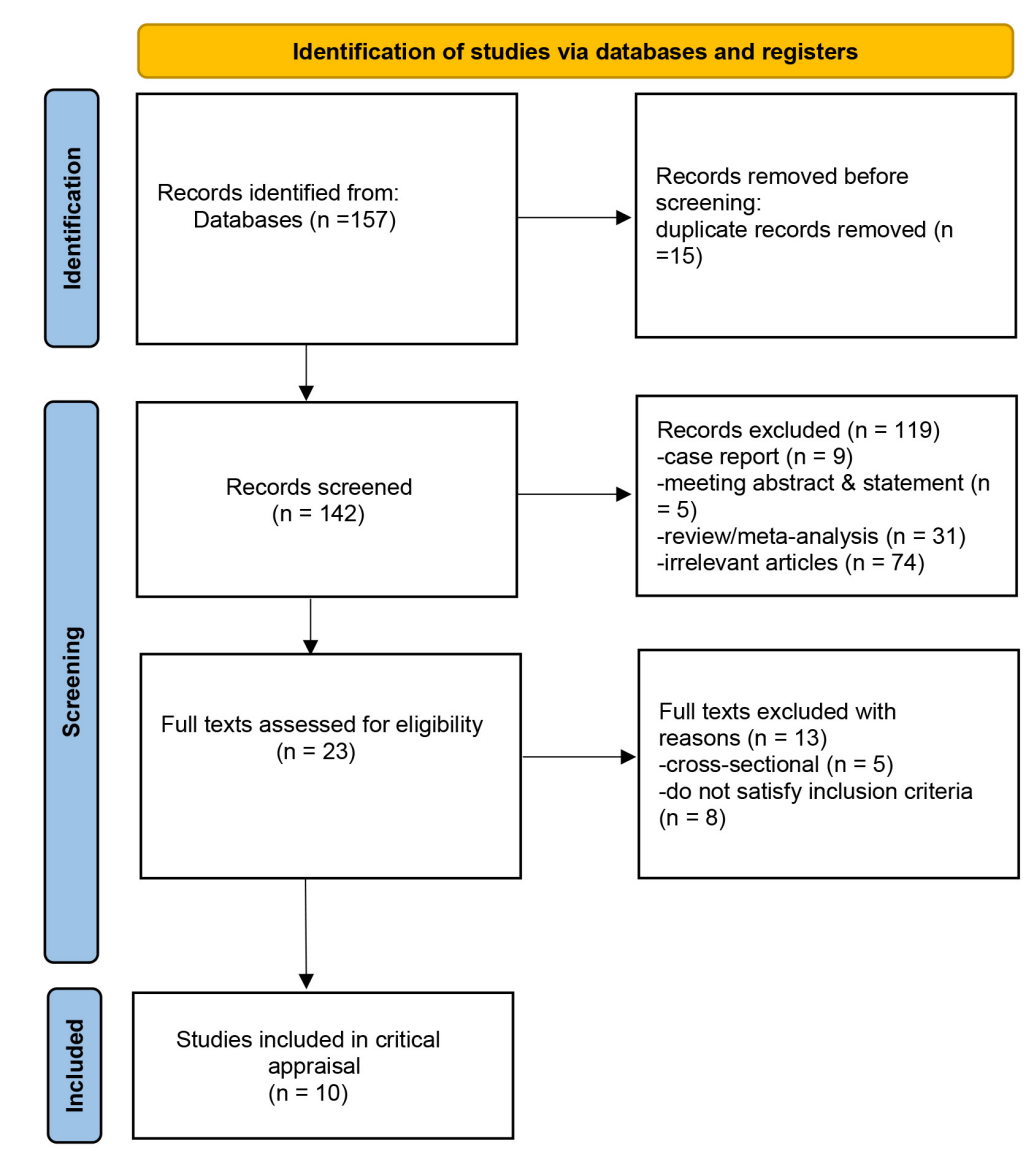
**Flowchart of study selection**.

We conducted a systematic review and meta-analysis following the guidelines outlined in the Preferred Reporting Items for Systematic Reviews and Meta-Analyses (PRISMA) to ensure comprehensive and transparent reporting of our methods and findings (Fig. 1).

### 2.1 Search Strategy

A comprehensive literature search was performed to locate all pertinent publications examining the impact of risk factors on in-hospital mortality among CHIPs. An electronic search was conducted using EMBASE, PubMed, Cochrane Library, Web of Science, Chinese Biomedical Database (CBM), China National Knowledge Infrastructure (CNKI), Wanfang databases, and China Science and Technology Journal Database (VIP) without any restrictions on publication date, sex, or ethnicity. The full search syntax used for PubMed is detailed in **Supplementary Material 1**. Additionally, the reference lists of all identified studies were manually reviewed to uncover any other relevant citations that the initial search may have missed.

### 2.2 Inclusion of Gray Literature

To ensure comprehensiveness, the search strategy included not only peer-reviewed articles but also gray literature such as conference abstracts and unpublished data. This was done to minimize the risk of publication bias and to include all potentially relevant data in the analysis.

### 2.3 Inclusion and Exclusion Criteria

#### 2.3.1 Inclusion Criteria

(1) Study Population: Articles were included if the study population included adult CHIPs after PCI supported by ECMO.

(2) Study Design: Cohort studies reporting on in-hospital mortality were included.

(3) Risk Factors: Risk factors of interest were evaluated, including body mass index (BMI), cardiogenic shock (CS) or cardiac arrest (CA) to ECMO, ECMO duration, type of infarction (e.g., left anterior descending (LAD)), lactate levels, left ventricular ejection fraction (LVEF), and mean arterial pressure (MAP). These indicators were chosen based on their biological and clinical relevance to cardiovascular outcomes and their known association with increased mortality risk in similar patient populations.

#### 2.3.2 Rationale for Selecting Specific Indicators

BMI: BMI is a widely used measure of body fat and has been consistently linked to various health outcomes, including cardiovascular disease. Higher BMI values are associated with an increased risk of in-hospital mortality due to the presence of multiple comorbidities such as diabetes, hypertension, and dyslipidemia, which can exacerbate the condition of CHIPs.

CS or CA to ECMO: The time from CS or CA to the initiation of ECMO support is critical. A longer duration between CS or CA and ECMO initiation is associated with higher mortality rates, as it indicates a longer period of ischemia and hypoxia, which can lead to irreversible organ damage and death.

ECMO Duration: The duration of ECMO support is inversely related to the risk of in-hospital mortality. A shorter ECMO duration suggests inadequate support for cardiac and pulmonary recovery, increasing the risk of mortality. Conversely, a longer ECMO duration allows for more extended support, potentially improving outcomes by providing sufficient time for organ recovery.

Type of Infarction (e.g., LAD): The type of infarction, particularly involving the LAD artery, is a significant predictor of in-hospital mortality. The LAD supplies a large area of the myocardium, and occlusions in this artery can lead to extensive myocardial damage, affecting cardiac function and prognosis. LAD infarctions are associated with higher mortality rates due to the severity of the myocardial damage.

Lactate Levels: Elevated lactate levels are indicative of tissue hypoxia and metabolic disturbance. High lactate levels reflect the severity of ischemia and are associated with poor outcomes, including increased in-hospital mortality. Monitoring lactate levels can provide insights into the effectiveness of interventions such as ECMO in restoring adequate tissue oxygenation.

LVEF: LVEF is a well-established indicator of cardiac function and prognosis in patients with cardiovascular disease. Lower LVEF values are associated with higher mortality rates due to impaired cardiac contractility.

MAP: MAP is a critical measure of circulatory stability. Hypotension, indicated by low MAP, can lead to inadequate organ perfusion and increased risk of mortality.

#### 2.3.3 Exclusion Criteria

(1) Non-CHIPs Controls: Articles were excluded if they compared the in-hospital mortality risk of CHIPs with (healthy) controls not CHIPs.

(2) Incomplete Data: Studies were excluded if the risk of death in CHIPs could not be obtained or if the abstract or full text were not available.

(3) Missing Data or Incomplete Mortality Reports: Studies with missing data or incomplete mortality reports were excluded to ensure the accuracy and reliability of the analysis. However, this exclusion criterion may lead to selection bias, as it could disproportionately exclude studies with poorer outcomes or higher mortality rates. The potential impact of such exclusions on the analysis results should be considered and discussed in the context of the study’s findings.

#### 2.3.4 Potential Impact of Exclusions

Excluding studies with missing data or incomplete mortality reports may introduce selection bias, as these studies might have different characteristics or outcomes compared to those included in the analysis. This bias could potentially skew the results towards more favorable outcomes or those with more complete data. It is important to acknowledge this limitation and consider its implications when interpreting the study’s findings.

### 2.4 Assessing Publication Bias

Possible publication bias was estimated by visual inspection of the funnel plots. To assess the possible impact of data from individual trials on the overall results, a sensitivity analysis was performed using a sequential leave-one-out analysis. We computed odds ratios (ORs) and confidence interval (CI) using a random-effects model when the studies had significant heterogeneity; otherwise, a fixed-effects model was selected. Results were considered statistically significant with *p* < 0.05.

### 2.5 Assessing the Risk of Bias

Two investigators (WJQ. and WYC.) independently assessed the quality of the included studies using the Newcastle–Ottawa Scale. Another investigator (YSZ.) resolved any differences in quality assessment. The quality of non-randomized controlled studies was assessed based on the following criteria:

(1) Representativeness of the Exposed Cohort: This criterion evaluates whether the study population is representative of the general population of interest. A score of 1 is given if the cohort is representative.

(2) Selection of the Non-Exposed Cohort: This criterion assesses the method of selecting the control group. A score of 1 is given if the control group is selected from the same population as the exposed cohort.

(3) Ascertainment of Exposure: This criterion evaluates the accuracy and reliability of the exposure measurement. A score of 1 is given if the exposure is clearly defined and measured.

(4) Outcome of Interest: This criterion assesses whether the study outcome is clearly defined and measured. A score of 1 is given if the outcome is clearly defined and measured.

(5) Comparability of Cohorts on Important Confounders: This criterion evaluates whether the study accounts for important confounders. A score of 1 is given if the study adjusts for at least one important confounder.

(6) Assessment of Outcome: This criterion assesses the method of outcome assessment. A score of 1 is given if the outcome is assessed in a valid and reliable manner.

(7) Length of Follow-Up: This criterion assesses the duration of the follow-up period. A score of 1 is given if the follow-up period is long enough to capture meaningful changes in the outcome of interest, ensuring that the study results are not biased by a short follow-up period.

(8) Adequacy of Follow-Up: This criterion evaluates whether the follow-up period is sufficient to observe the outcome of interest. A score of 1 is given if the follow-up is adequate.

#### 2.5.1 Scoring Criteria for Quality Assessment

Moderate Quality (Score: 5–7): Studies that meet most of the criteria but have some limitations in design or execution are considered of moderate quality.

High Quality (Score: 8–9): Studies that meet all or nearly all of the criteria with minimal limitations are considered high quality.

#### 2.5.2 Detailed Scoring Process for Each Study

The detailed scoring process for each study is presented in Table 1 (Ref. [6, 7, 8, 9, 10, 11, 12, 13, 14, 15]). Each study was evaluated based on the criteria listed above, and the total score was calculated. The quality of each study was then classified as moderate or high based on the total score. Eight observational studies were of moderate quality (total score: 5–7) and two studies were of high quality (total score: 8). Overall, the comparability between the groups was fair.

**Table 1.** **Quality assessment**.

	Selection					Outcome			
Study	Representativeness	Non exposed cohort	Ascertainment of exposure	Outcome of interest	Comparability	Assessment of outcome	Length of follow-up	Adequacy of follow-up	Total Score
Loskutov *et al*. [6] 2020	+	+	—	+	+	+	+	+	7
Rigamonti *et al*. [7] 2016	+	+	—	+	++	+	+	+	8
Pang *et al*. [8] 2022	+	—	—	+	++	+	+	+	7
Zumuletti *et al*. [9] 2017	+	—	—	—	+	+	+	+	5
Wu *et al*. [10] 2018	+	—	—	+	—	+	+	+	5
Fu *et al*. [11] 2017	+	+	—	+	+	+	+	+	7
Liang *et al*. [12] 2021	+	—	—	+	+	+	+	+	6
Pan *et al*. [13] 2022	+	+	—	+	++	+	+	+	8
Liu *et al*. [14] 2019	+	—	—	+	+	—	+	+	5
Xie *et al*. [15] 2021	+	—	—	+	—	+	+	+	5

Assessment with “+” is a score of 1, “—” is not scored. The total score of 9, less than five is low quality research, 5–7 is moderate quality research, 8–9 is high quality research.

### 2.6 Data Extraction and Analysis

Following the selection of articles for inclusion, we extracted key information including the first author, publication year, study design, data collection year, study population characteristics (such as sample size, mean age, and sex distribution), diagnostic criteria for CHIPs, risk factors being investigated, sources of comorbidity information, number of deaths, and duration of follow-up. When studies presented data on various factors affecting the time of survival, we focused solely on the risk factors associated with in-hospital mortality.

A meta-analysis was conducted to assess the nature and extent of the associations between risk factors and the outcomes under investigation. For each analysis, we employed the effect estimates for individual risk factors as documented in the original publications. Most of the included studies supplied sample sizes for these risk factors. When data were insufficient, we reached out to the authors for further details. Studies were omitted from the analysis if they could not supply the necessary data or if there was no response from the authors.

All statistical analyses were conducted utilizing the Cochrane Review Manager software (RevMan 5.4.1; The Nordic Cochrane Centre, The Cochrane Collaboration, Copenhagen, Denmark, 2020). Pooled odds ratios (ORs) and mean differences (MDs) were calculated, complete with 95% confidence intervals (CIs), for both categorical and continuous data sets. The estimation of mean and standard deviation (SD) followed the methodologies detailed by McGrath *et al*. [16]. In instances where continuous data were presented as median ± interquartile range (IQR), these estimation techniques were appropriately applied.

### 2.7 Heterogeneity Assessment and Management

Heterogeneity among the studies was assessed using the I^2^ statistic. An I^2^ index ≥50% indicated significant heterogeneity among the studies. Given the significant heterogeneity observed in some analyses, a sensitivity analysis was performed to manage this heterogeneity.

Sensitivity analyses were conducted by sequentially excluding individual studies to assess their impact on the overall results. This approach helped to identify studies that may have contributed significantly to the observed heterogeneity. By excluding each study one at a time, we were able to determine the stability of the pooled estimates and identify any potential outliers that could have influenced the results. These studies may have had different methodological approaches, patient populations, or data collection methods, which could have influenced the overall results. By excluding these studies, we were able to obtain more stable and reliable estimates of the associations between the risk factors and in-hospital mortality.

## 3. Results

### 3.1 Study Selection and Characteristics

The preliminary search identified 157 relevant articles. We removed 15 duplicates. Following the evaluation of abstracts, the application of our inclusion and exclusion criteria, and an assessment of bias risk, we were left with ten studies [6, 7, 8, 9, 10, 11, 12, 13, 14, 15] (Fig. 1 and Table 2 (Ref. [6, 7, 8, 9, 10, 11, 12, 13, 14, 15])). A flowchart of the selection process is shown in Fig. 1. To date, no randomized trials have been conducted. All studies were cohort studies [6, 7, 8, 9, 10, 11, 12, 13, 14, 15]. A total of 306 patients (100%) were treated with ECMO.

**Table 2.** **Baseline characteristics of the included studies**.

Author	Year	Country/District	Non-survivors (n)	Survivors (n)	Observed Indexes	Follow-up Duration	Interventions
Loskutov *et al*. [6]	2020	Ukraine	13	10	①②③④⑤⑥⑧⑩⑪	30 days	PCI + ECMO
Rigamonti *et al*. [7]	2016	Switzerland	18	11	①②③④⑤⑥⑦⑧⑩⑬	30 days	PCI + ECMO
Pang *et al*. [8]	2022	China	21	19	①②③④⑤⑥⑧⑨⑩⑪	30 days	PCI + ECMO
Zumuletti *et al*. [9]	2017	China	9	10	①②④⑤⑥⑦⑧	60 days	PCI + ECMO
Wu *et al*. [10]	2018	China	20	17	①②③④⑤⑥⑦⑧⑨⑩⑪⑫⑬	30 days	PCI + ECMO
Fu *et al*. [11]	2017	China	15	12	①②③④⑤⑥⑦⑧⑨⑩⑪⑫⑬	60 days	PCI + ECMO
Liang *et al*. [12]	2017	China	24	19	①②③④⑤⑥⑦⑧⑨⑩⑪⑫⑬	60 days	PCI + ECMO
Pan *et al*. [13]	2022	China	16	6	①②③④⑤⑥⑦⑧⑨⑩⑪⑫	30 days	PCI + ECMO
Liu *et al*. [14]	2019	China	8	6	①②⑦⑧⑩⑪	30 days	PCI + ECMO
Xie *et al*. [15]	2021	China	17	35	①②③④⑤⑥⑧⑨⑩⑪	30 days	PCI + ECMO

Notes: ① = Age; ② = Male; ③ = Body mass index (BMI); ④ = Smoking history; ⑤ = Hypertension; ⑥ = Diabetes mellitus; ⑦ = Cardiogenic shock (CS) or cardiac arrest (CA) to extracorporeal membrane oxygenation (ECMO); ⑧ = ECMO duration; ⑨ = Type of infarction-left anterior descending (LAD), left circumflex (LCX), right coronary artery (RCA), left main coronary artery (LMCA); ⑩ = Lactate; ⑪ = Left ventricular ejection fraction (LVEF); ⑫ = Mean arterial pressure (MAP); ⑬ = Heart rate. Follow-up duration: duration of follow-up for each study. Interventions: types of interventions provided, such as ECMO, percutaneous coronary intervention (PCI).

The study characteristics and baseline patient demographics are presented in Table 2. ECMO alone was used as the intervention. The mean age was >60 years old. All ten studies used Veno-arterial (V/A) ECMO oxygenation. A total of 306 patients across all studies underwent PCI. Most patients were male (70.3%). Eight studies [6, 7, 8, 10, 11, 12, 13, 15] referred to BMI. Nine studies [6, 7, 8, 9, 10, 11, 12, 13, 15] reported smoking history, hypertension, and diabetes mellitus. Seven studies [7, 9, 10, 11, 12, 13, 14] reported on CS or CA to ECMO.

Ten studies [6, 7, 8, 9, 10, 11, 12, 13, 14, 15] included ECMO duration. Seven studies [6, 8, 10, 11, 12, 13, 15] reported the type of infarction: LAD, left circumflex (LCX), right coronary artery (RCA), and left main coronary artery (LMCA). Nine studies [6, 7, 8, 10, 11, 12, 13, 14, 15] mentioned lactate levels, while eight [6, 8, 10, 11, 12, 13, 14, 15] referred to LVEF. Finally, four [10, 11, 12, 13] referred to MAP, and another four studies [7, 10, 11, 12] reported heart rate (Table 2).

### 3.2 Determinants

Many studies have offered a comprehensive examination of various factors influencing the survival rates of CHIPs, focusing primarily on statistically significant multivariate ORs. Consequently, the calculation of both univariate and multivariate pooled effect estimates for each determinant was conducted solely when these factors were explored in a minimum of three studies. The multivariate effect estimates are visually represented through forest plots.

### 3.3 Population Characteristics

**Fig. 2.**
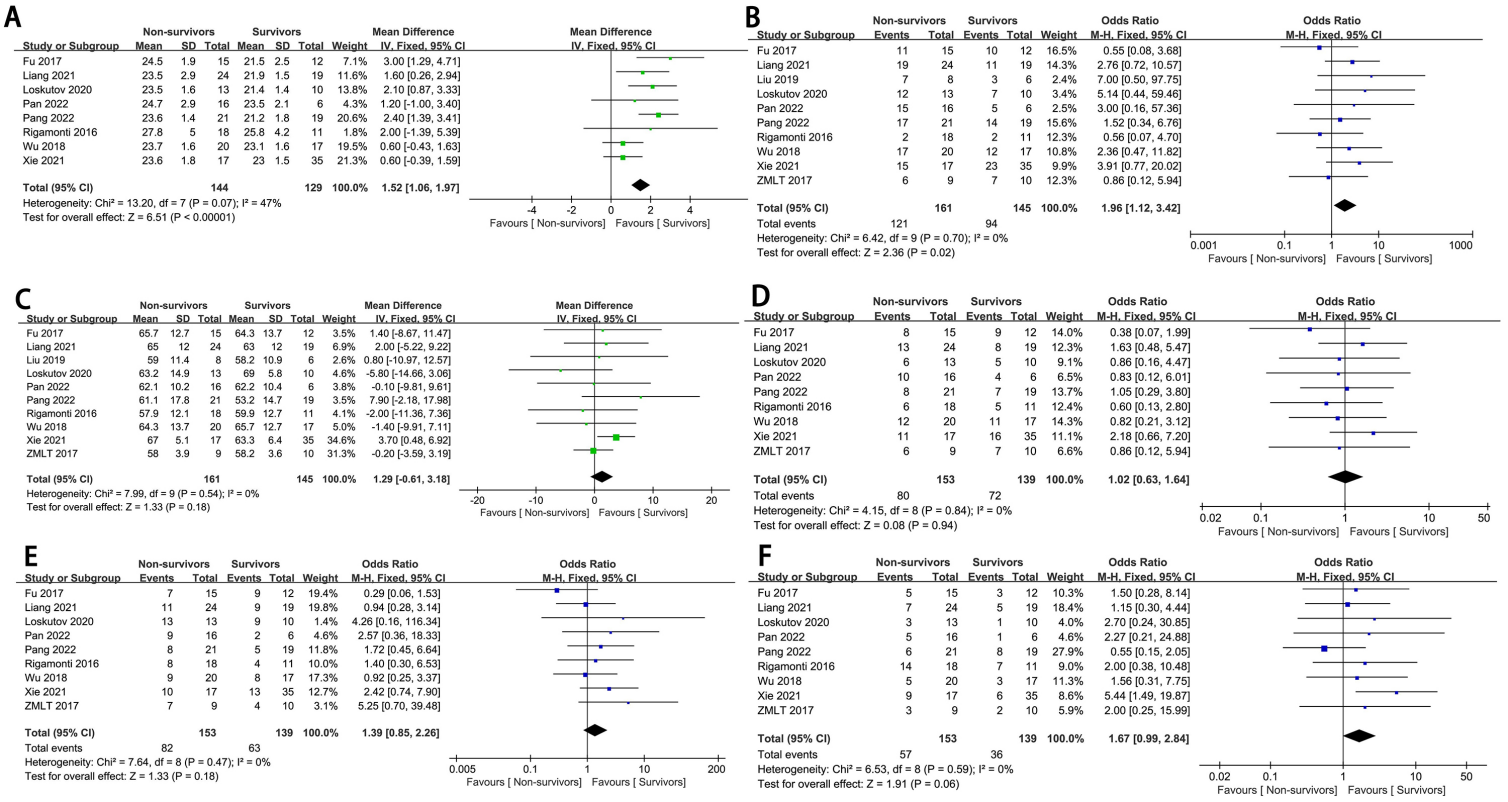
**The forest plot of population characteristics as high-risk factors for in-hospital mortality in complex high-risk and indicated patients (CHIPs)**. (A) BMI. (B) Male. (C) Age. (D) Smoking history. (E) Hypertension. (F) Diabetes mellitus. SD, standard deviation; IV, inverse variance; M-H, mantel-haenszel method.

Patients that were male (OR = 1.96, 95% CI: 1.12 to 3.42, *p* = 0.02, I^2^ = 0%) or obese (mean difference, MD:1.52, 95% CI: 1.06 to 1.97, *p* < 0.00001, I^2^ = 47%) had higher in-hospital mortality, which was not age related. Additionally, underlying medical conditions such as smoking history, hypertension, and diabetes, were not related to in-hospital mortality (Fig. 2).

### 3.4 ECMO Related Content

#### 3.4.1 CS or CA to ECMO

**Fig. 3.**
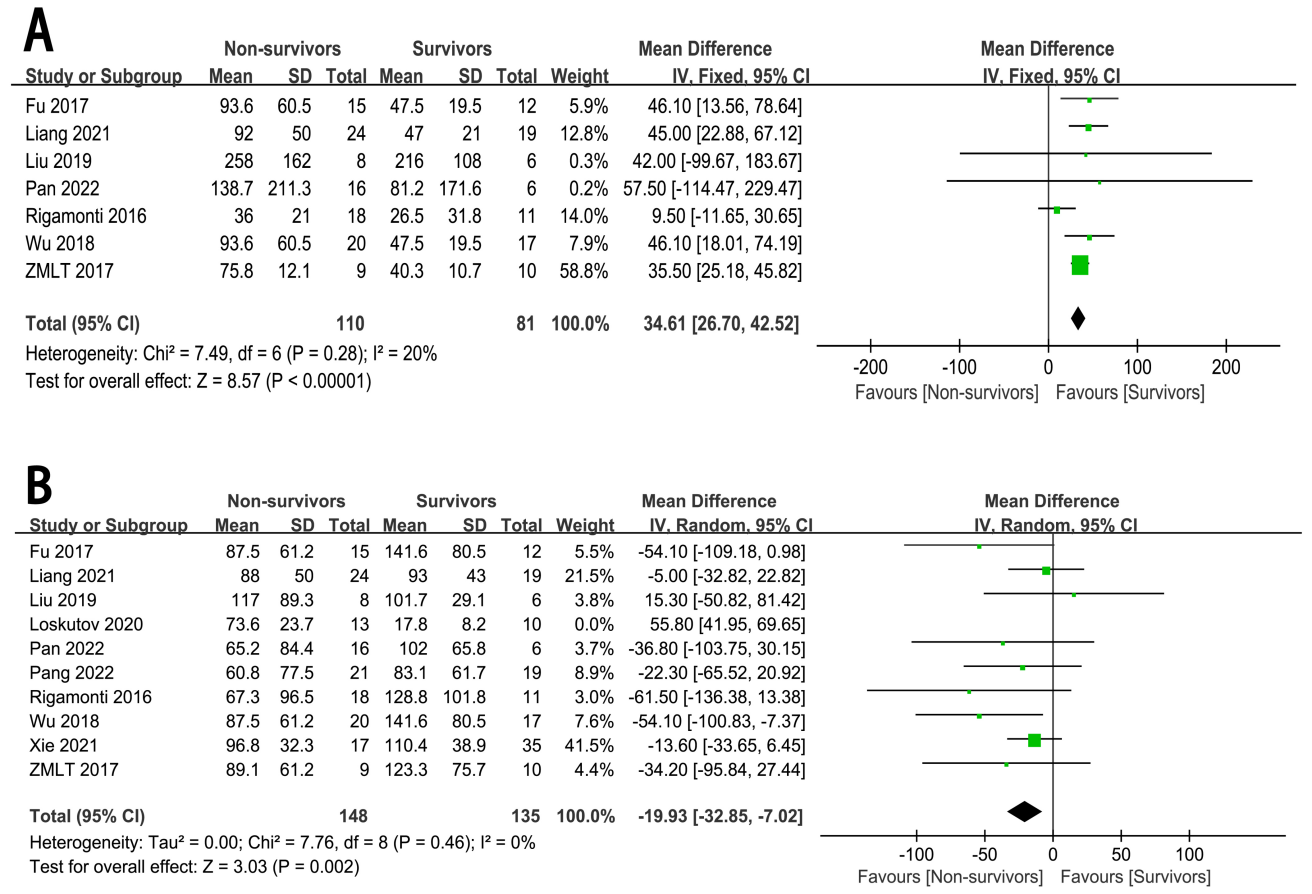
**The forest plot of ECMO related content as high-risk factors for in-hospital mortality in CHIPs**. (A) CS or CA to ECMO. (B) ECMO duration.

Seven studies [7, 9, 10, 11, 12, 13, 14] examined the association between CS or CA to ECMO and the risk of in-hospital mortality (n = 191 participants/110 deaths). The summary effect size for in-hospital mortality, comparing the longer and shorter CS or CA to ECMO, was 34.61 (95% CI: 26.70 to 42.52, *p* < 0.00001, I^2^ = 20%), indicating a significant positive association between CS or CA to ECMO and in-hospital mortality (Fig. 3). The shorter CS or CA to ECMO reduced the incidence of in-hospital mortality.

#### 3.4.2 ECMO Duration

In view of the significant heterogeneity among the studies (I^2^ = 87%, *p* < 0.00001), sensitivity analysis was performed. After the exclusion of the study by Loskutov *et al*. [6], a total of 283 patients were included, which resulted in a decreased heterogeneity (I^2^ = 0%, *p* = 0.46). The pooled effect size for in-hospital mortality, when comparing prolonged versus brief ECMO duration, was –19.93 (95% Cl: –32.85 to –7.02, *p* = 0.002). This suggests a significant inverse correlation between the duration of ECMO support and the risk of in-hospital mortality (Fig. 3). Therefore, a longer duration of ECMO can decrease the incidence of in-hospital mortality.

### 3.5 Coronary Artery Vascular Conditions

#### Type of Infarction

**Fig. 4.**
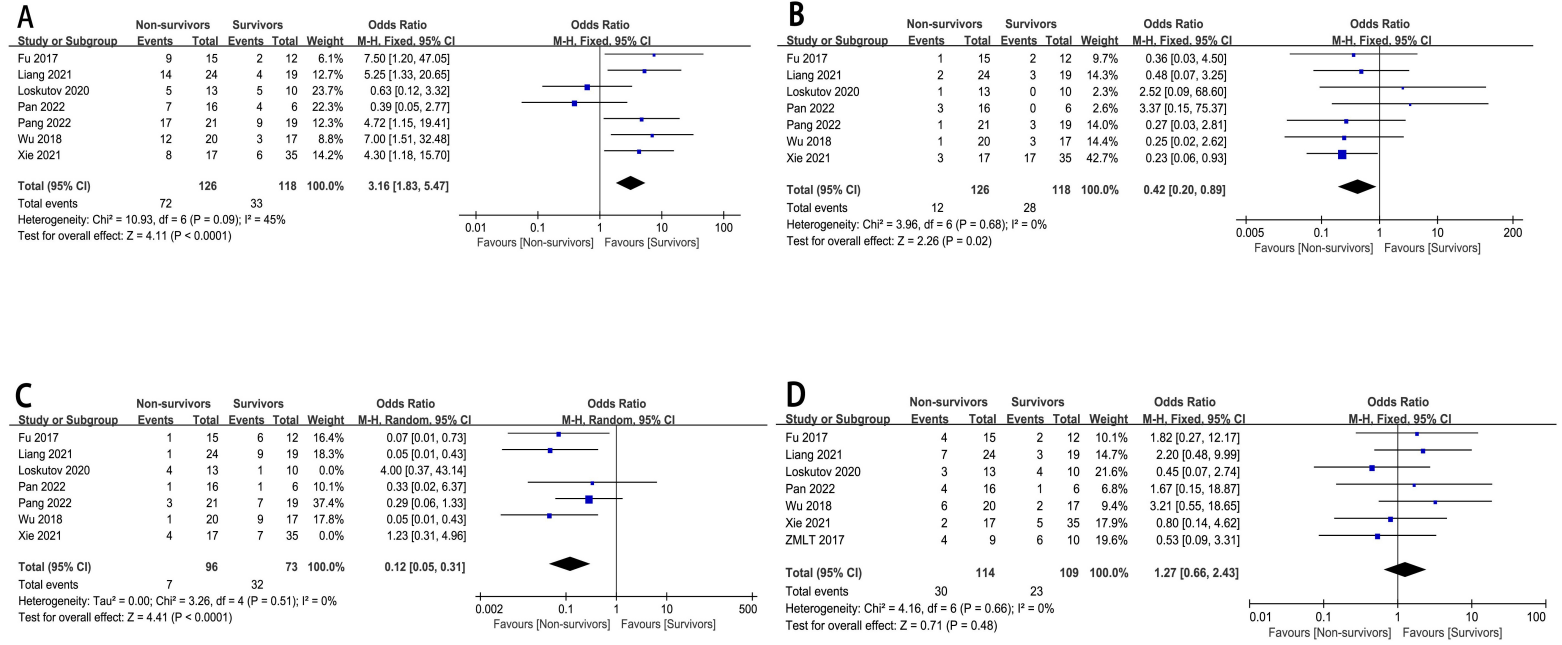
**The forest plot of coronary artery vascular conditions as high-risk factors for in-hospital mortality in CHIPs**. (A) Type of infarction-LAD. (B) Type of infarction-LCX. (C) Type of infarction-RCA. (D) Type of infarction-LMCA.

The relationship between the type of infarction (LAD, LCX, RCA, LMCA) and in-hospital mortality risk has been well described (n = 7) [6, 8, 10, 11, 12, 13, 15], in 244 patients. The pooled analysis revealed a statistically significantly greater in-hospital mortality risk in CHIPs with LAD (OR = 3.16, 95% CI : 1.83 to 5.47, *p* < 0.0001, I^2^ = 45%), LCX (OR = 0.42, 95% CI : 0.20 to 0.89, *p* = 0.02, I^2^ = 0%), and RCA (OR = 0.12, 95% CI : 0.05 to 0.31, *p* < 0.0001) infarctions, while no such effect was observed for LMCA (Fig. 4). The chi-square test results indicated that LAD vascular lesions were more significantly associated with the risk of death in CHIPs than LCX or RCA lesions (**Supplementary Material 5 **). There was considerable heterogeneity in the results among the seven studies (RCA) (I^2^ = 61%, *p* = 0.02). A sensitivity analysis was conducted. After excluding the study by Loskutov *et al*. [6] and Xie *et al*. [15], 169 patients were included, resulting in decreased heterogeneity (RCA; I^2^ = 0%, *p* = 0.51).

### 3.6 Biochemical and Inspection Indicators

**Fig. 5.**
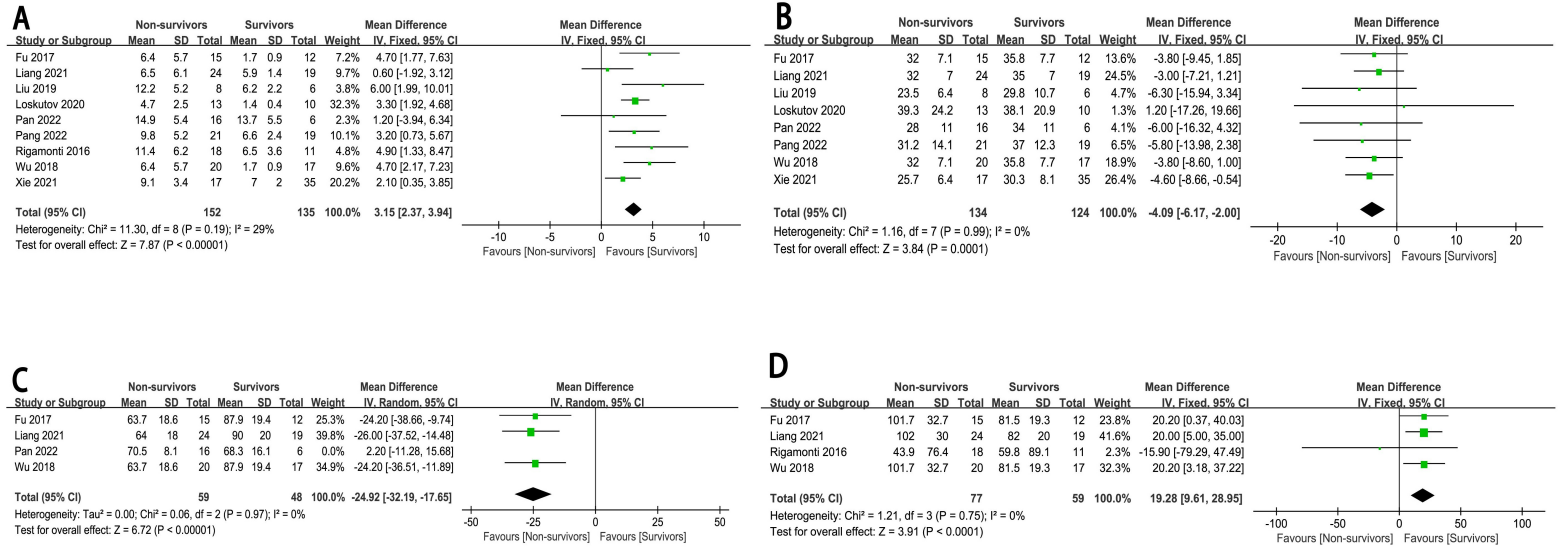
**The forest plot of biochemical and inspection indicators as high-risk factors for in-hospital mortality in CHIPs**. (A) Lactate. (B) LVEF. (C) MAP. (D) Heart rate.

Our research findings indicated that patients with higher lactate levels (MD: 3.15, 95% CI: 2.37 to 3.94, *p* < 0.00001, I^2^ = 29%) and heart rate (MD: 19.28, 95% CI: 9.61 to 28.95, *p* < 0.0001, I^2^ = 0%), but lower LVEF (MD: –4.09, 95% CI: –6.17 to –2.00, *p* = 0.0001, I^2^ = 0%), and MAP (MD: –24.92, 95% CI: –32.19 to –17.65, *p* < 0.00001, I^2^ = 0%), had a higher risk of in-hospital mortality (Fig. 5). There was considerable heterogeneity in the results among the four studies [6, 13, 14, 15] (MAP: I^2^ = 75%, *p* = 0.15). For MAP, excluding the study by Pan *et al*. [13], resulted in 107 patients being included, decreasing heterogeneity (I^2^ = 0%, *p* = 0.97).

## 4. Discussion

### 4.1 Research Summary

The main finding of this study was the identification of several ECMO-related content risk factors (CS or CA to ECMO, ECMO duration), coronary artery vascular conditions risk factors (type of infarction), population demographic risk factors (BMI, male), and biochemical and inspection indicator risk factors (lactate levels, LVEF, MAP, heart rate) that are associated with an increased risk for mortality in CHIPs. CS or CA to ECMO, lactate levels, and heart rate significantly correlated with mortality, while ECMO duration, LVEF, and MAP had negative correlations. There was a very strong correlation between CS or CA to ECMO, ECMO duration, type of infarction (LAD), and in-hospital mortality in CHIPs. Conversely, some risk factors that are typically linked to a poor prognosis in patients did not show a significant association with an increased risk of in-hospital mortality in CHIPs. These factors include the type of infarction involving the LMCA, age, smoking history, hypertension, and diabetes mellitus.

### 4.2 High-risk Factors

#### 4.2.1 CS or CA to ECMO and ECMO Duration

Most studies suggest that the longer the CS or CA to ECMO, the higher the in-hospital mortality in CHIPs; however, the opposite is true for the duration of ECMO. ECMO can provide near-normal cerebral and end-organ perfusion [3, 17]. The ability to provide full cerebral and end-organ blood supply, for days or weeks, with ECMO has enabled a paradigm shift in cardiac arrest—preservation of the brain while awaiting the return of spontaneous circulation (ROSC), definitive care, and cardiac recovery [4]. Therefore, the longer the duration between CS or CA to ECMO, the longer the brain and end organs of the patient are subjected to ischemia and hypoxia, resulting in a higher mortality rate. Similarly, the shorter the ECMO duration, the lower the blood supply to the heart and brain.

#### 4.2.2 Type of Infarction-LAD

LAD infarction was found to be associated with higher mortality rates. LAD infarction is particularly high-risk due to the artery’s critical role in supplying blood to a large area of the myocardium. Occlusions in the LAD can lead to extensive myocardial damage, affecting cardiac function and prognosis. The hemodynamic consequences of LAD stenosis are significant. As the stenosis increases from 60 to 70%, there is a dramatic change in hemodynamics, with a significant pressure difference and increased wall shear stress observed at the site of the stenosis. This increase in wall shear stress, along with changes in blood flow velocity, can exacerbate myocardial ischemia [18].

Furthermore, the recirculation zone in the post-stenotic region can contribute to the formation of additional stenoses, further complicating blood flow and increasing the risk of ischemia [19]. These hemodynamic alterations are crucial in understanding why LAD infarctions are associated with higher mortality rates.

While the literature shows some variability on the impact of LAD infarction, there is a consistent trend indicating that patients with LAD infarction face a higher risk of in-hospital mortality compared to infarcts in other territories (**Supplementary Material 5**).

#### 4.2.3 BMI

A high BMI was associated with a higher in-hospital mortality risk in CHIPs. The prevalence of obesity is increasing worldwide, with ~20% of intensive care unit (ICU) patients reported to be obese [20]. Adipose tissue is highly metabolically active, and visceral adipose tissue has a deleterious adipocyte secretory profile, resulting in insulin resistance and a chronic low-grade inflammatory and procoagulant state [20]. Obesity is strongly associated with chronic diseases, including type 2 diabetes, hypertension, cardiovascular diseases, dyslipidemia, non-alcoholic fatty liver disease, chronic kidney disease, obstructive sleep apnea and hypoventilation syndrome, mood disorders, and physical disabilities [20]. In hospitalized and ICU patients and in patients with chronic illnesses, a J-shaped relationship between BMI and in-hospital mortality has been demonstrated [20]. This may be related to the increased cardiovascular risk factors and more severe conditions of patients with a high BMI. In addition, a high BMI can also affect the safety and effectiveness of ECMO cannulation. Patients with a higher BMI have additional complications during cannulation of the femoral vessels for ECMO. Anatomical variations in obese patients can make blood vessels more difficult to access, increasing the technical and operational challenges during the cannulation process. Moreover, excessive subcutaneous fat may increase the risk of infection, as surgical incisions may be more difficult to keep sterile, and the fat layer could become a breeding ground for bacteria. Therefore, for patients with a higher BMI, when performing femoral vessel cannulation, physicians should carefully assess the patient’s anatomical structure and vascular conditions, and take appropriate preventive measures to reduce the occurrence of these complications.

#### 4.2.4 Lactate, LVEF, MAP, Heart Rate

Our results showed that lactate levels, LVEF, MAP, and heart rate are increased risk factors, which may be due to the fact that most CHIPs are in a state of stress. An excessive heart rate and lactic acid production can lead to increased myocardial oxygen consumption and a short diastolic period. When the LVEF and MAP are excessively low, coronary perfusion is reduced, and systemic hemodynamic changes occur, all of which contributes to increased mortality [21]. The initial serum lactate levels and age are independent predictors of in-hospital mortality. Elevated lactate levels are a critical indicator of systemic hypoperfusion and tissue hypoxia. Lactate is produced during anaerobic metabolism when oxygen supply to tissues is inadequate. High levels of lactate reflect a state of tissue hypoxia, where cells are forced to rely on anaerobic glycolysis for energy production due to a limited supply of oxygen. This shift to anaerobic metabolism results in the accumulation of lactate, which can be measured in the blood as an indirect marker of tissue oxygenation [22]. Systemic hypoperfusion, often resulting from conditions such as shock or severe heart failure, leads to reduced blood flow to vital organs and tissues. This reduction in perfusion exacerbates tissue hypoxia and triggers a cascade of metabolic derangements. Metabolic disturbances, including acidosis and electrolyte imbalances, can further impair cellular function and contribute to organ dysfunction [22].

Patients presenting with hypotension (MAP <65 mmHg) upon admission to the ICU have a significantly higher 28-day in-hospital mortality rate than those without hypotension [23]. Therefore, clinicians should carefully assess these risk factors and consider them in treatment plans to improve patient prognosis.

With the rapid development of mechanical assistive device therapy, ECMO has gradually become a treatment option for CHIPs after PCI. ECMO supports life by using extracorporeal equipment to replace or support lung and heart function to enhance cardiac and pulmonary recovery [4]. This study demonstrated that shorter CS or CA to ECMO and longer ECMO duration, can significantly reduce the in-hospital mortality of CHIPs, improve their prognosis, and reduce the incidence of risk factors associated with increased mortality.

### 4.3 Comparison with Existing Literature

Our study contributes to the existing literature by specifically addressing gaps in the understanding of ECMO’s impact on post-PCI mortality in CHIPs. While previous meta-analyses have examined the role of ECMO in various clinical settings, none have exclusively targeted its effect on in-hospital mortality following PCI in this patient population. Our study fills this gap by identifying independent high-risk factors associated with increased in-hospital mortality among CHIPs after PCI with ECMO support.

Compared to other meta-analyses, our study provides a more focused analysis on the specific risk factors relevant to CHIPs, such as cardiogenic shock or cardiac arrest to ECMO time, ECMO duration, and type of infarction. This targeted approach allows for a deeper understanding of the factors influencing mortality in this high-risk group, offering valuable insights that can guide clinical decision-making and improve patient outcomes.

### 4.4 Impact of Heterogeneity and Study Quality

The heterogeneity observed among the studies, with an I^2^ index ≥50% in some analyses, indicates significant variability in the effect sizes across studies. This heterogeneity may stem from differences in study populations, methodologies, and data collection techniques. To manage this heterogeneity, we performed sensitivity analyses by sequentially excluding individual studies to assess their impact on the overall results. This approach helped identify studies that contributed significantly to the observed heterogeneity and allowed us to obtain more stable and reliable estimates of the associations between the risk factors and in-hospital mortality.

The quality of the included studies was assessed using the Newcastle–Ottawa Scale, with studies classified as moderate or high quality based on their total scores. The quality of evidence directly impacts the reliability of the results obtained from the meta-analysis. By including only studies that met specific quality criteria, we aimed to minimize bias and ensure the robustness of our findings.

### 4.5 Additional Considerations

The lack of individual participant data (IPD) in our study introduces potential confounding bias, as we are unable to control for all possible confounders at the individual level. This limitation could impact the accuracy of our risk factor associations. Additionally, the small sample size of the included studies may reduce statistical power, limiting our ability to detect smaller but potentially meaningful effects.

### 4.6 Suggestions for Addressing These Issues

To address these issues, future research should aim to collect and analyze IPD to allow for more granular control of confounders and improve the precision of risk factor associations. Additionally, larger sample sizes in future studies will increase statistical power and enable the detection of smaller effect sizes. Finally, a more detailed explanation and analysis of sensitivity analysis results, particularly for low-quality studies, should be provided to ensure a comprehensive understanding of the data’s robustness and reliability.

In summary, our study’s unique contributions lie in its focused approach to examining ECMO’s impact on post-PCI mortality in CHIPs and its comprehensive analysis of heterogeneity and study quality to ensure the results’ validity and reliability. Addressing these identified limitations in future research will further strengthen the field’s understanding of ECMO’s role in CHIPs.

### 4.7 Limitations

This study had certain limitations. First, the meta-analysis was a secondary analysis; thus, defects of the included studies impact the reliability of the results of the meta-analysis. Additionally, the lack of individual participant data precluded a more detailed analysis of prognosis or the estimation of individual patient outcomes. In addition, a certain degree of heterogeneity was observed among the studies, with a possibility of bias. Therefore, it is necessary to further verify the results of this study using larger samples and greater homogeneity. Future research should focus on validating the risk assessment tools identified in this study within multicenter prospective studies. This approach will help to confirm the generalizability and reliability of these tools across different clinical settings and patient populations. By conducting multicenter studies, researchers can account for variations in patient care and patient demographics, thereby strengthening the validity of the risk assessment models. There is a need for studies that investigate the impact of dynamic lactate monitoring and targeted interventions on patient outcomes. Given the association between elevated lactate levels and mortality identified in our study, understanding how real-time lactate monitoring can guide clinical interventions is crucial. Future studies should explore how changes in lactate levels over time can inform treatment decisions and improve patient survival rates.

In addition, this study did not have a registered protocol. The study was initiated based on the urgency to address a clinical question without the foresight of protocol registration, which may impact the generalizability of our findings. Future studies in this area should aim to prospectively register their protocols to enhance the transparency and credibility of the research process.

Meanwhile, our study’s generalizability is somewhat limited by the predominance of regional data sources, which may not reflect global variations in CHIPs outcomes following PCI with ECMO support. The concentrated geographic focus could introduce biases, affecting the universality of our conclusions. Future studies should expand their scope to include a more diverse range of geographic regions to capture a broader spectrum of patient experiences and healthcare practices. This will help in developing more comprehensive and globally applicable treatment guidelines for CHIPs.

Finally, as a meta-analysis, our study relies on data from existing literature, which may limit our ability to quantitatively assess the impact of specific risk factors. The main purpose of a meta-analysis is to qualitatively evaluate the association between risk factors and in-hospital mortality, rather than to quantitatively predict the exact percentage increase in risk. Therefore, although we can confirm the significant association between risk factors and increased in-hospital mortality, we cannot precisely quantify the extent of this increased risk.

## 5. Conclusions

In this study, we conducted a comprehensive analysis of multiple observational studies to explore the risk factors associated with in-hospital mortality in CHIPs following PCI supported by ECMO. Our findings reveal several key risk factors that significantly negatively impact the in-hospital mortality risk in CHIPs.

First, we found that a prolonged duration from CS or CA to the initiation of ECMO support is significantly associated with an increased risk of in-hospital mortality in CHIPs. This suggests that the timely initiation of ECMO support is crucial for improving outcomes in CHIPs. Delayed ECMO initiation may lead to hypoxia and ischemia in vital organs, thereby increasing the risk of mortality.

Second, the duration of ECMO support was found to be inversely related to the risk of in-hospital mortality. Specifically, a shorter duration of ECMO use is associated with a higher risk of in-hospital mortality. This may reflect the need for CHIPs to have more time under ECMO support to recover cardiac function and hemodynamic stability. Extended ECMO support may provide patients with additional opportunities to recover, thereby reducing the risk of mortality.

Additionally, our study identified that LAD infarction is associated with an increased risk of in-hospital mortality in CHIPs. The LAD supplies a large area of the myocardium, and LAD lesions may lead to more extensive myocardial damage, affecting cardiac function and prognosis.

We also observed that a higher BMI is associated with an increased risk of in-hospital mortality in CHIPs. High BMI may be linked to various cardiovascular risk factors, including diabetes, hypertension, and dyslipidemia, which may act in concert to increase the mortality risk in CHIPs.

Elevated serum lactate levels were also identified as an independent predictor of in-hospital mortality in CHIPs. Increased lactate levels reflect the severity of tissue hypoxia and metabolic disturbance, which may be associated with poor outcomes in CHIPs.

Finally, lower LVEF and MAP were associated with an increased risk of in-hospital mortality in CHIPs. These decreased hemodynamic parameters may reflect impaired cardiac contractility and circulatory failure in CHIPs, both of which are strong predictors of in-hospital mortality.

In summary, our study results emphasize the importance of timely ECMO initiation, optimizing the duration of ECMO support, identifying and managing LAD infarction, controlling BMI, and maintaining hemodynamic stability in reducing the risk of in-hospital mortality of CHIPs. These findings provide clinicians with more precise risk assessment tools to devise more effective treatment plans and improve the prognosis of CHIPs.

## Data Availability

The data that support the findings of this study are available from the corresponding author upon reasonable request.

## Abbreviations

AMI, acute myocardial infarction; CHIPs, complex high-risk and indicated patients; CS, cardiogenic shock; CA, cardiac arrest; CS or CA-ECMO, time from CS or CA to ECMO device implantation; ECMO, extracorporeal membrane oxygenation; LAD, left anterior descending; LCX, left circumflex; LMCA, left main coronary artery; LVEF, left ventricle ejection fraction; MAP, mean article pressure; PCI, percutaneous coronary intervention; RCA, right coronary artery.

## Supplementary Material

Supplementary material associated with this article can be found, in the online version, at https://doi.org/10.31083/RCM27126.
