# Supplementary material for: High-Risk Factors of In-Hospital Death Following Complex High-risk and Indicated Patients After Percutaneous Coronary Intervention Supported by Extracorporeal Membrane Oxygenation
Source: Rev Cardiovasc Med. 2025 May 26;26(5):27126. doi: 10.31083/RCM27126 (PMC12135673; doi:10.31083/RCM27126)
Supplement: Supplementary file 1 [file 2153-8174-26-5-27126-s1.zip › Supplementary Material 3 Results/Results1.docx]

| *Outcomes* | *Non-survivors* | *Survivors* | *Mean Difference (IV, 95% CI)* | *P value* | *I^2^* | *Z* |
| --- | --- | --- | --- | --- | --- | --- |
| **ECMO related content** |  |  |  |  |  |  |
| ECMO duration | 148 | 135 | -19.93[-32.85，-7.02] | 0.002 | 0% | 3.03 |
| CS or CA to ECMO | 110 | 81 | 34.61 [26.70, 42.52] | <0.00001 | 20% | 8.57 |
| **Population characteristics** |  |  |  |  |  |  |
| Age | 161 | 145 | 1.29 [-0.61, 3.18] | 0.18 | 0% | 1.33 |
| BMI | 144 | 129 | 1.52 [1.06, 1.97] | <0.00001 | 47% | 6.51 |
| **Biochemical and Inspection Indicators** |  |  |  |  |  |  |
| Lactate | 152 | 135 | 3.15 [2.37, 3.94] | <0.00001 | 29% | 7.87 |
| LVEF | 134 | 124 | -4.09 [-6.17, -2.00] | 0.0001 | 0% | 3.84 |
| MAP | 59 | 48 | -24.92 [-32.19,-17.65] | <0.00001 | 0% | 6.72 |
| heart rate | 77 | 59 | 19.28 [9.61, 28.95] | < 0.0001 | 0% | 3.91 |
