# Supplementary material for: High-Risk Factors of In-Hospital Death Following Complex High-risk and Indicated Patients After Percutaneous Coronary Intervention Supported by Extracorporeal Membrane Oxygenation
Source: Rev Cardiovasc Med. 2025 May 26;26(5):27126. doi: 10.31083/RCM27126 (PMC12135673; doi:10.31083/RCM27126)
Supplement: Supplementary file 1 [file 2153-8174-26-5-27126-s1.zip › Supplementary Material 3 Results/Results2.docx]

| *Outcomes* | *Non-survivors* | *Survivors* | *Odds Ratio (M-H, 95% CI)* | *P value* | *I^2^* | *Z* |
| --- | --- | --- | --- | --- | --- | --- |
| **Coronary artery vascular conditions** |  |  |  |  |  |  |
| Type of infarction-associated CA-LAD | 72/126 | 33/118 | 3.16 [1.83, 5.47] | < 0.0001 | 45% | 4.11 |
| Type of infarction-associated CA-LM | 30/114 | 23/109 | 1.27 [0.66, 2.43] | 0.48 | 0% | 0.71 |
| Type of infarction-associated CA-LCX | 12/126 | 28/118 | 0.42 [0.20, 0.89] | 0.02 | 0% | 2.26 |
| Type of infarction-associated CA-RCA | 7/96 | 32/73 | 0.12 [0.05, 0.31] | < 0.0001 | 0% | 4.85 |
| **Population characteristics** |  |  |  |  |  |  |
| Male | 121/161 | 94/145 | 1.96 [1.12, 3.42] | 0.02 | 0% | 2.36 |
| Smoking history | 80/153 | 72/139 | 1.02 [0.63, 1.64] | 0.94 | 0% | 0.08 |
| hypertension | 82/153 | 63/139 | 1.39 [0.85, 2.26] | 0.18 | 0% | 1.33 |
| Diabetes mellitus | 57/153 | 36/139 | 1.67 [0.99, 2.84] | 0.06 | 0% | 1.91 |
