# Supplementary material for: High-Risk Factors of In-Hospital Death Following Complex High-risk and Indicated Patients After Percutaneous Coronary Intervention Supported by Extracorporeal Membrane Oxygenation
Source: Rev Cardiovasc Med. 2025 May 26;26(5):27126. doi: 10.31083/RCM27126 (PMC12135673; doi:10.31083/RCM27126)
Supplement: Supplementary file 1 [file 2153-8174-26-5-27126-s1.zip › Supplementary Material 5 Doc 1. Chi-Square(LAD, LCX, RCA).doc]

WEIGHT BY frequency.
CROSSTABS
  /TABLES=CA BY outcome BY frequency
  /FORMAT=AVALUE TABLES
  /STATISTICS=CHISQ
  /CELLS=COUNT ROW
  /COUNT ROUND CELL.


Crosstabulation


Case Processing Summary	
	Cases	
	Valid	Missing	Total	
	N	Percent	N	Percent	N	Percent	
CA * outcome * frequency	200	100.0%	0	0.0%	200	100.0%	


CA * outcome * frequency Crosstabulation	
frequency	outcome	Total	
	death	survival		
12.00	CA	LCX	Count	12		12	
			% within CA	100.0%		100.0%	
	Total	Count	12		12	
		% within CA	100.0%		100.0%	
15.00	CA	RCA	Count	15		15	
			% within CA	100.0%		100.0%	
	Total	Count	15		15	
		% within CA	100.0%		100.0%	
28.00	CA	LCX	Count		28	28	
			% within CA		100.0%	100.0%	
	Total	Count		28	28	
		% within CA		100.0%	100.0%	
33.00	CA	LAD	Count		33	33	
			% within CA		100.0%	100.0%	
	Total	Count		33	33	
		% within CA		100.0%	100.0%	
40.00	CA	RCA	Count		40	40	
			% within CA		100.0%	100.0%	
	Total	Count		40	40	
		% within CA		100.0%	100.0%	
72.00	CA	LAD	Count	72		72	
			% within CA	100.0%		100.0%	
	Total	Count	72		72	
		% within CA	100.0%		100.0%	
Total	CA	LAD	Count	72	33	105	
			% within CA	68.6%	31.4%	100.0%	
		LCX	Count	12	28	40	
			% within CA	30.0%	70.0%	100.0%	
		RCA	Count	15	40	55	
			% within CA	27.3%	72.7%	100.0%	
	Total	Count	99	101	200	
		% within CA	49.5%	50.5%	100.0%	


Chi-Square Tests	
frequency	Value	df	Asymptotic Significance (2-sided)	
12.00	Pearson Chi-Square	.b			
	N of Valid Cases	12			
15.00	Pearson Chi-Square	.b			
	N of Valid Cases	15			
28.00	Pearson Chi-Square	.b			
	N of Valid Cases	28			
33.00	Pearson Chi-Square	.b			
	N of Valid Cases	33			
40.00	Pearson Chi-Square	.b			
	N of Valid Cases	40			
72.00	Pearson Chi-Square	.b			
	N of Valid Cases	72			
Total	Pearson Chi-Square	32.233a	2	.000	
	Likelihood Ratio	33.193	2	.000	
	N of Valid Cases	200			

a. 0 cells (00.0%) have expected count less than 5. The minimum expected count is 19.80.	
b. No statistics are computed because CA and outcome are constants.	

USE ALL.
COMPUTE filter_$=(coronary = 1 | coronary = 2).
VARIABLE LABELS filter_$ 'coronary = 1 | coronary = 2 (FILTER)'.
VALUE LABELS filter_$ 0 'Not Selected' 1 'Selected'.
FORMATS filter_$ (f1.0).
FILTER BY filter_$.
EXECUTE.
CROSSTABS
  /TABLES=CA BY outcome BY frequency
  /FORMAT=AVALUE TABLES
  /STATISTICS=CHISQ
  /CELLS=COUNT ROW
  /COUNT ROUND CELL.


Crosstabulation


Case Processing Summary	
	Cases	
	Valid	Missing	Total	
	N	Percent	N	Percent	N	Percent	
CA * outcome * frequency	145	100.0%	0	0.0%	145	100.0%	


CA * outcome * frequency Crosstabulation	
frequency	outcome	Total	
	death	survival		
12.00	CA	LCX	Count	12		12	
			% within CA	100.0%		100.0%	
	Total	Count	12		12	
		% within CA	100.0%		100.0%	
28.00	CA	LCX	Count		28	28	
			% within CA		100.0%	100.0%	
	Total	Count		28	28	
		% within CA		100.0%	100.0%	
33.00	CA	LAD	Count		33	33	
			% within CA		100.0%	100.0%	
	Total	Count		33	33	
		% within CA		100.0%	100.0%	
72.00	CA	LAD	Count	72		72	
			% within CA	100.0%		100.0%	
	Total	Count	72		72	
		% within CA	100.0%		100.0%	
Total	CA	LAD	Count	72	33	105	
			% within CA	68.6%	31.4%	100.0%	
		LCX	Count	12	28	40	
			% within CA	30.0%	70.0%	100.0%	
	Total	Count	84	61	145	
		% within CA	57.9%	42.1%	100.0%	


Chi-Square Tests	
frequency	Value	df	Asymptotic Significance (2-sided)	Exact Sig. (2-sided)	
12.00	Pearson Chi-Square	.c				
	N of Valid Cases	12				
28.00	Pearson Chi-Square	.c				
	N of Valid Cases	28				
33.00	Pearson Chi-Square	.c				
	N of Valid Cases	33				
72.00	Pearson Chi-Square	.c				
	N of Valid Cases	72				
Total	Pearson Chi-Square	17.682a	1	.000		
	Continuity Correctionb	16.135	1	.000		
	Likelihood Ratio	17.758	1	.000		
	Fisher's Exact Test				.000	
	N of Valid Cases	145				

Chi-Square Tests	
frequency	Exact Sig. (1-sided)	
12.00	Pearson Chi-Square		
	N of Valid Cases		
28.00	Pearson Chi-Square		
	N of Valid Cases		
33.00	Pearson Chi-Square		
	N of Valid Cases		
72.00	Pearson Chi-Square		
	N of Valid Cases		
Total	Pearson Chi-Square		
	Continuity Correctionb		
	Likelihood Ratio		
	Fisher's Exact Test	.000	
	N of Valid Cases		

a. 0 cells (00.0%) have expected count less than 5. The minimum expected count is 16.83.	
b. Computed only for a 2x2 table	
c. No statistics are computed because CA and outcome are constants.	

USE ALL.
COMPUTE filter_$=(coronary = 1 | coronary = 3).
VARIABLE LABELS filter_$ 'coronary = 1 | coronary = 3 (FILTER)'.
VALUE LABELS filter_$ 0 'Not Selected' 1 'Selected'.
FORMATS filter_$ (f1.0).
FILTER BY filter_$.
EXECUTE.
CROSSTABS
  /TABLES=CA BY outcome BY frequency
  /FORMAT=AVALUE TABLES
  /STATISTICS=CHISQ
  /CELLS=COUNT ROW
  /COUNT ROUND CELL.


Crosstabulation


Case Processing Summary	
	Cases	
	Valid	Missing	Total	
	N	Percent	N	Percent	N	Percent	
CA * outcome * frequency	160	100.0%	0	0.0%	160	100.0%	


CA * outcome * frequency Crosstabulation	
frequency	outcome	Total	
	death	survival		
15.00	CA	RCA	Count	15		15	
			% within CA	100.0%		100.0%	
	Total	Count	15		15	
		% within CA	100.0%		100.0%	
33.00	CA	LAD	Count		33	33	
			% within CA		100.0%	100.0%	
	Total	Count		33	33	
		% within CA		100.0%	100.0%	
40.00	CA	RCA	Count		40	40	
			% within CA		100.0%	100.0%	
	Total	Count		40	40	
		% within CA		100.0%	100.0%	
72.00	CA	LAD	Count	72		72	
			% within CA	100.0%		100.0%	
	Total	Count	72		72	
		% within CA	100.0%		100.0%	
Total	CA	LAD	Count	72	33	105	
			% within CA	68.6%	31.4%	100.0%	
		RCA	Count	15	40	55	
			% within CA	27.3%	72.7%	100.0%	
	Total	Count	87	73	160	
		% within CA	54.4%	45.6%	100.0%	


Chi-Square Tests	
frequency	Value	df	Asymptotic Significance (2-sided)	Exact Sig. (2-sided)	
15.00	Pearson Chi-Square	.c				
	N of Valid Cases	15				
33.00	Pearson Chi-Square	.c				
	N of Valid Cases	33				
40.00	Pearson Chi-Square	.c				
	N of Valid Cases	40				
72.00	Pearson Chi-Square	.c				
	N of Valid Cases	72				
Total	Pearson Chi-Square	24.814a	1	.000		
	Continuity Correctionb	23.178	1	.000		
	Likelihood Ratio	25.403	1	.000		
	Fisher's Exact Test				.000	
	N of Valid Cases	160				

Chi-Square Tests	
frequency	Exact Sig. (1-sided)	
15.00	Pearson Chi-Square		
	N of Valid Cases		
33.00	Pearson Chi-Square		
	N of Valid Cases		
40.00	Pearson Chi-Square		
	N of Valid Cases		
72.00	Pearson Chi-Square		
	N of Valid Cases		
Total	Pearson Chi-Square		
	Continuity Correctionb		
	Likelihood Ratio		
	Fisher's Exact Test	.000	
	N of Valid Cases		

a. 0 cells (00.0%) have expected count less than 5. The minimum expected count is 25.09.	
b. Computed only for a 2x2 table	
c. No statistics are computed because CA and outcome are constants.	

USE ALL.
COMPUTE filter_$=(coronary = 2 | coronary = 3).
VARIABLE LABELS filter_$ 'coronary = 2 | coronary = 3 (FILTER)'.
VALUE LABELS filter_$ 0 'Not Selected' 1 'Selected'.
FORMATS filter_$ (f1.0).
FILTER BY filter_$.
EXECUTE.
CROSSTABS
  /TABLES=CA BY outcome BY frequency
  /FORMAT=AVALUE TABLES
  /STATISTICS=CHISQ
  /CELLS=COUNT ROW
  /COUNT ROUND CELL.


Crosstabulation


Case Processing Summary	
	Cases	
	Valid	Missing	Total	
	N	Percent	N	Percent	N	Percent	
CA * outcome * frequency	95	100.0%	0	0.0%	95	100.0%	


CA * outcome * frequency Crosstabulation	
frequency	outcome	Total	
	death	survival		
12.00	CA	LCX	Count	12		12	
			% within CA	100.0%		100.0%	
	Total	Count	12		12	
		% within CA	100.0%		100.0%	
15.00	CA	RCA	Count	15		15	
			% within CA	100.0%		100.0%	
	Total	Count	15		15	
		% within CA	100.0%		100.0%	
28.00	CA	LCX	Count		28	28	
			% within CA		100.0%	100.0%	
	Total	Count		28	28	
		% within CA		100.0%	100.0%	
40.00	CA	RCA	Count		40	40	
			% within CA		100.0%	100.0%	
	Total	Count		40	40	
		% within CA		100.0%	100.0%	
Total	CA	LCX	Count	12	28	40	
			% within CA	30.0%	70.0%	100.0%	
		RCA	Count	15	40	55	
			% within CA	27.3%	72.7%	100.0%	
	Total	Count	27	68	95	
		% within CA	28.4%	71.6%	100.0%	


Chi-Square Tests	
frequency	Value	df	Asymptotic Significance (2-sided)	Exact Sig. (2-sided)	
12.00	Pearson Chi-Square	.c				
	N of Valid Cases	12				
15.00	Pearson Chi-Square	.c				
	N of Valid Cases	15				
28.00	Pearson Chi-Square	.c				
	N of Valid Cases	28				
40.00	Pearson Chi-Square	.c				
	N of Valid Cases	40				
Total	Pearson Chi-Square	.085a	1	.771		
	Continuity Correctionb	.004	1	.952		
	Likelihood Ratio	.084	1	.771		
	Fisher's Exact Test				.820	
	N of Valid Cases	95				

Chi-Square Tests	
frequency	Exact Sig. (1-sided)	
12.00	Pearson Chi-Square		
	N of Valid Cases		
15.00	Pearson Chi-Square		
	N of Valid Cases		
28.00	Pearson Chi-Square		
	N of Valid Cases		
40.00	Pearson Chi-Square		
	N of Valid Cases		
Total	Pearson Chi-Square		
	Continuity Correctionb		
	Likelihood Ratio		
	Fisher's Exact Test	.474	
	N of Valid Cases		

a. 0 cells (00.0%) have expected count less than 5. The minimum expected count is 11.37.	
b. Computed only for a 2x2 table	
c. No statistics are computed because CA and outcome are constants.	

SET TLook=None FOOTNOTE=ON Small=0.0001 SUMMARY=None THREADS=AUTO SIGLESS=YES TFit=Both DIGITGROUPING=No LEADZERO=No TABLERENDER=light.
SET OLang=SChinese Unicode=Yes Locale=OSLOCALE Small=0.0001 THREADS=AUTO Printback=On SIGLESS=YES BASETEXTDIRECTION=AUTOMATIC DIGITGROUPING=No TLook=None FOOTNOTE=ON SUMMARY=None MIOUTPUT=[observed imputed pooled diagnostics] TFit=Both LEADZERO=No TABLERENDER=light.
