# Supplementary figures and images for: High-Risk Factors of In-Hospital Death Following Complex High-risk and Indicated Patients After Percutaneous Coronary Intervention Supported by Extracorporeal Membrane Oxygenation
Source: Rev Cardiovasc Med. 2025 May 26;26(5):27126. doi: 10.31083/RCM27126 (PMC12135673; doi:10.31083/RCM27126)

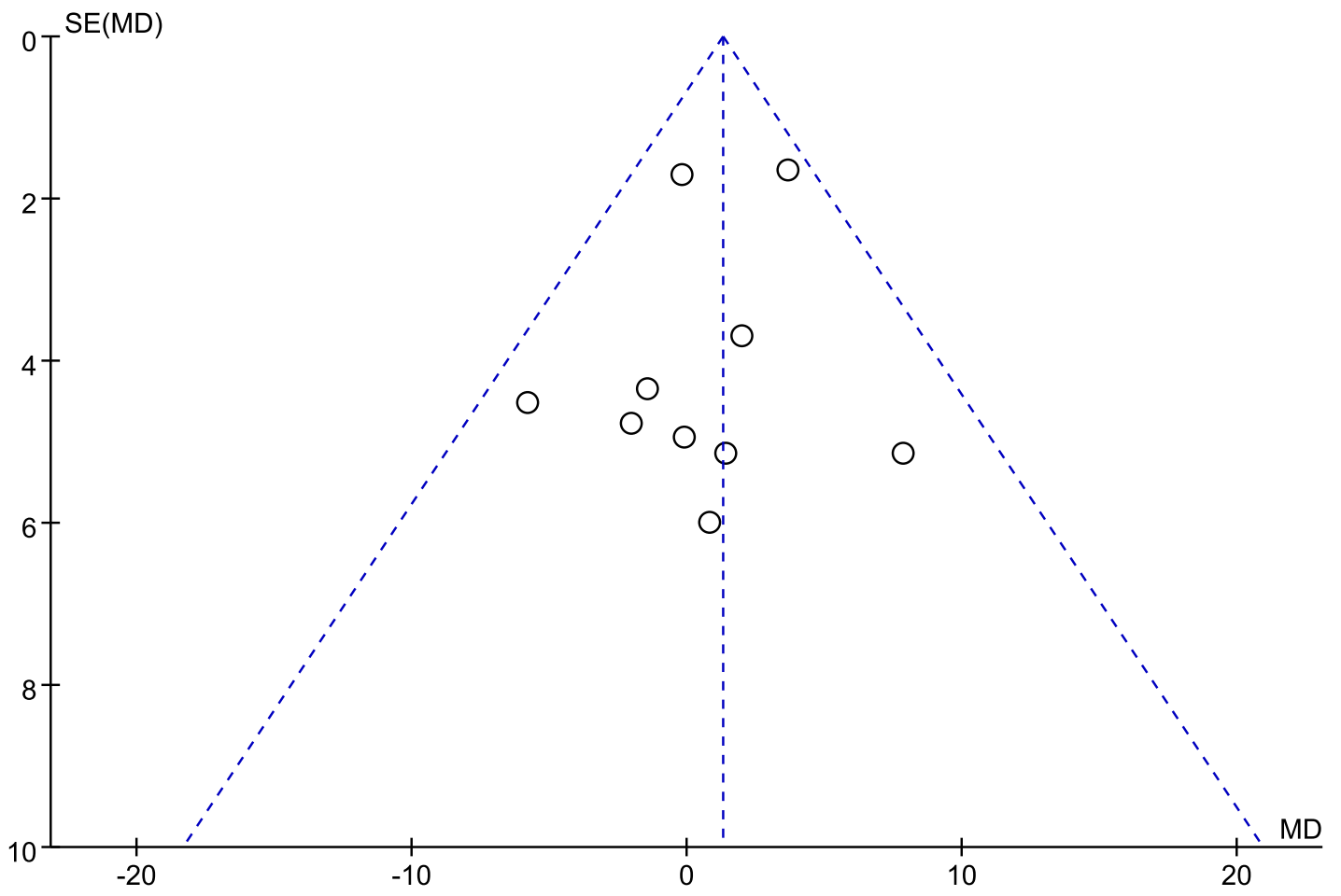

Supplement: Supplementary file 1 [file 2153-8174-26-5-27126-s1.zip › Supplementary Material 2 Funnel plot/Age Funnel plot.pdf]

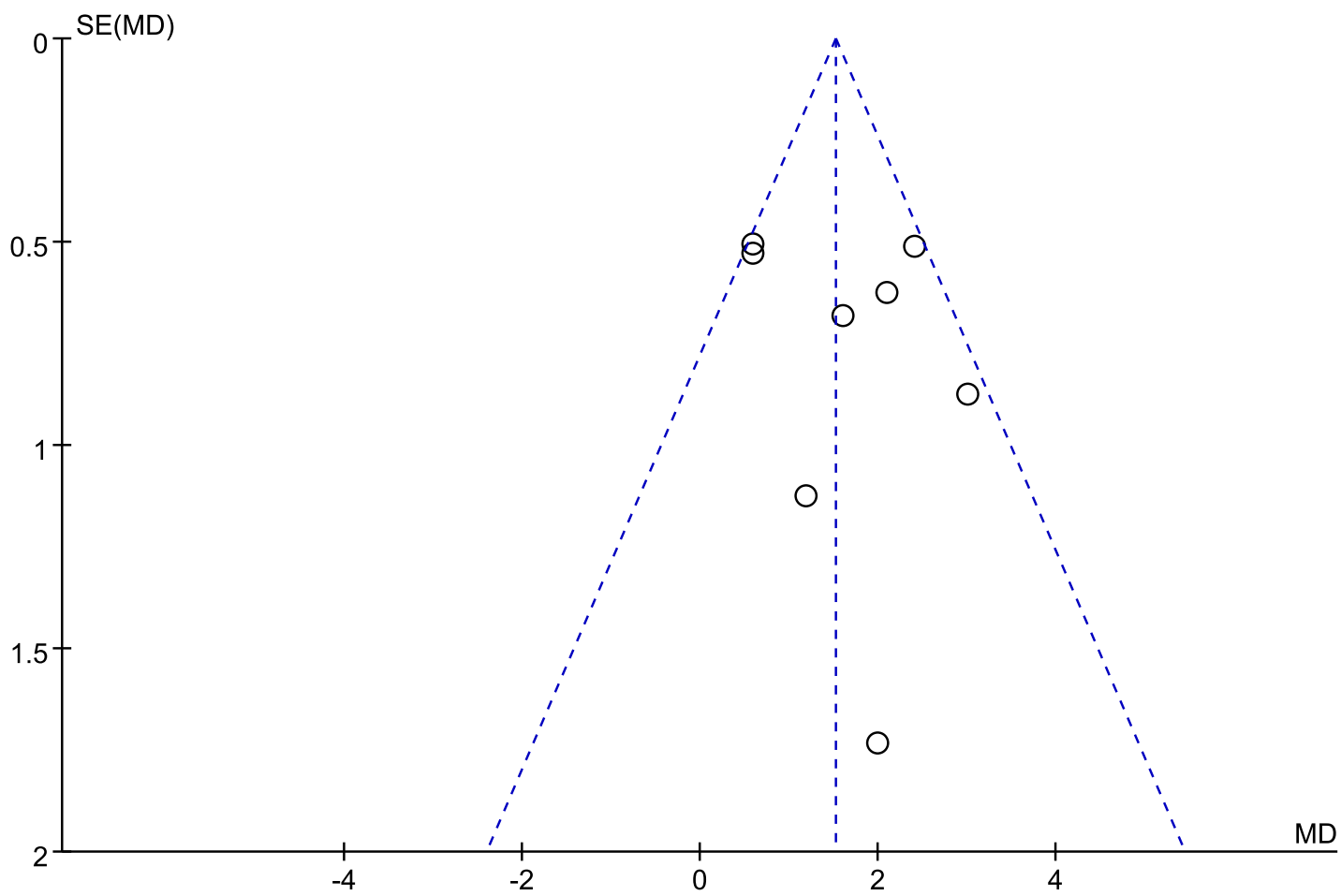

Supplement: Supplementary file 1 [file 2153-8174-26-5-27126-s1.zip › Supplementary Material 2 Funnel plot/BMI Funnel plot.pdf]

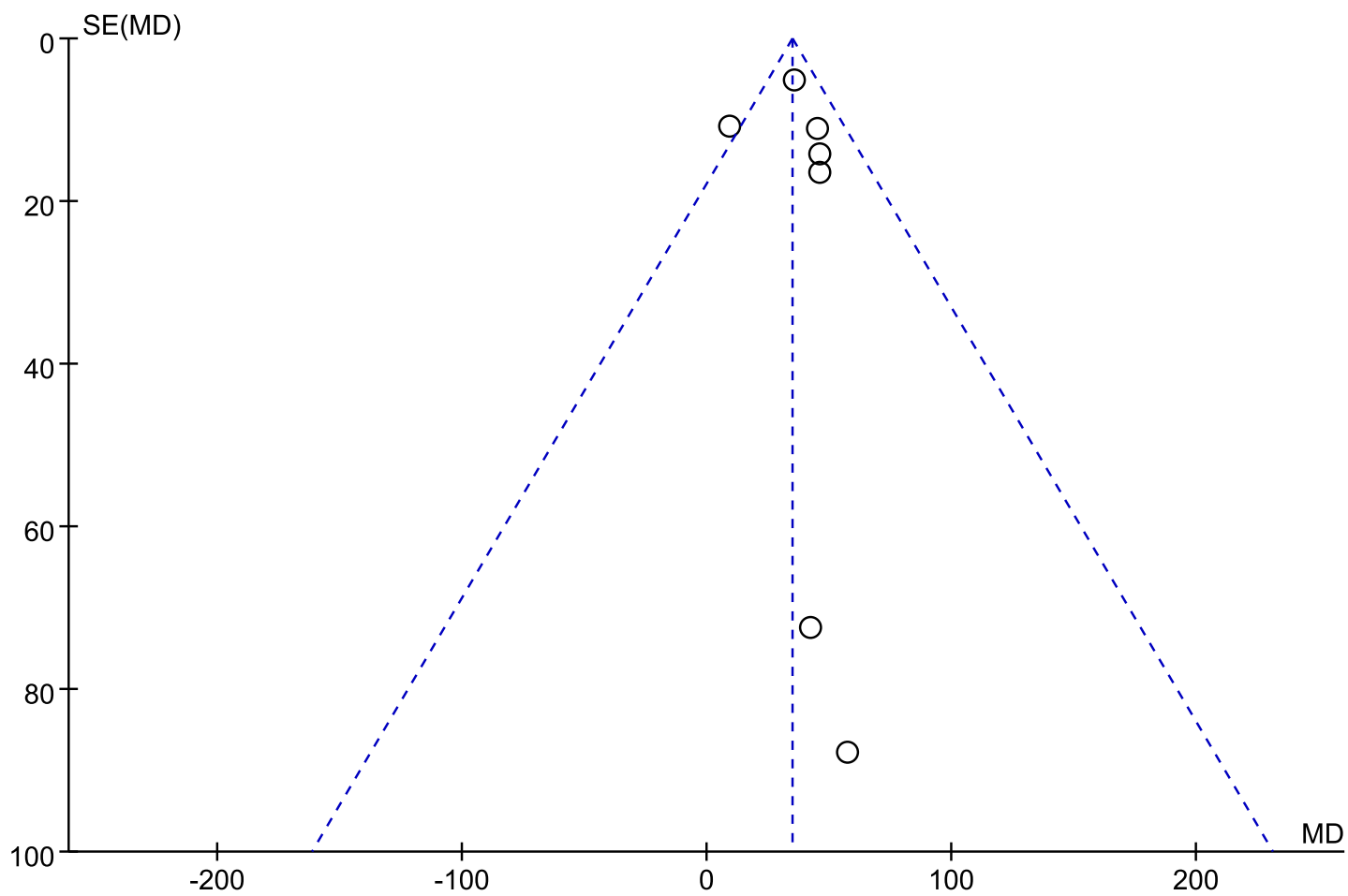

Supplement: Supplementary file 1 [file 2153-8174-26-5-27126-s1.zip › Supplementary Material 2 Funnel plot/CS or CA-ECMO Funnel plot.pdf]

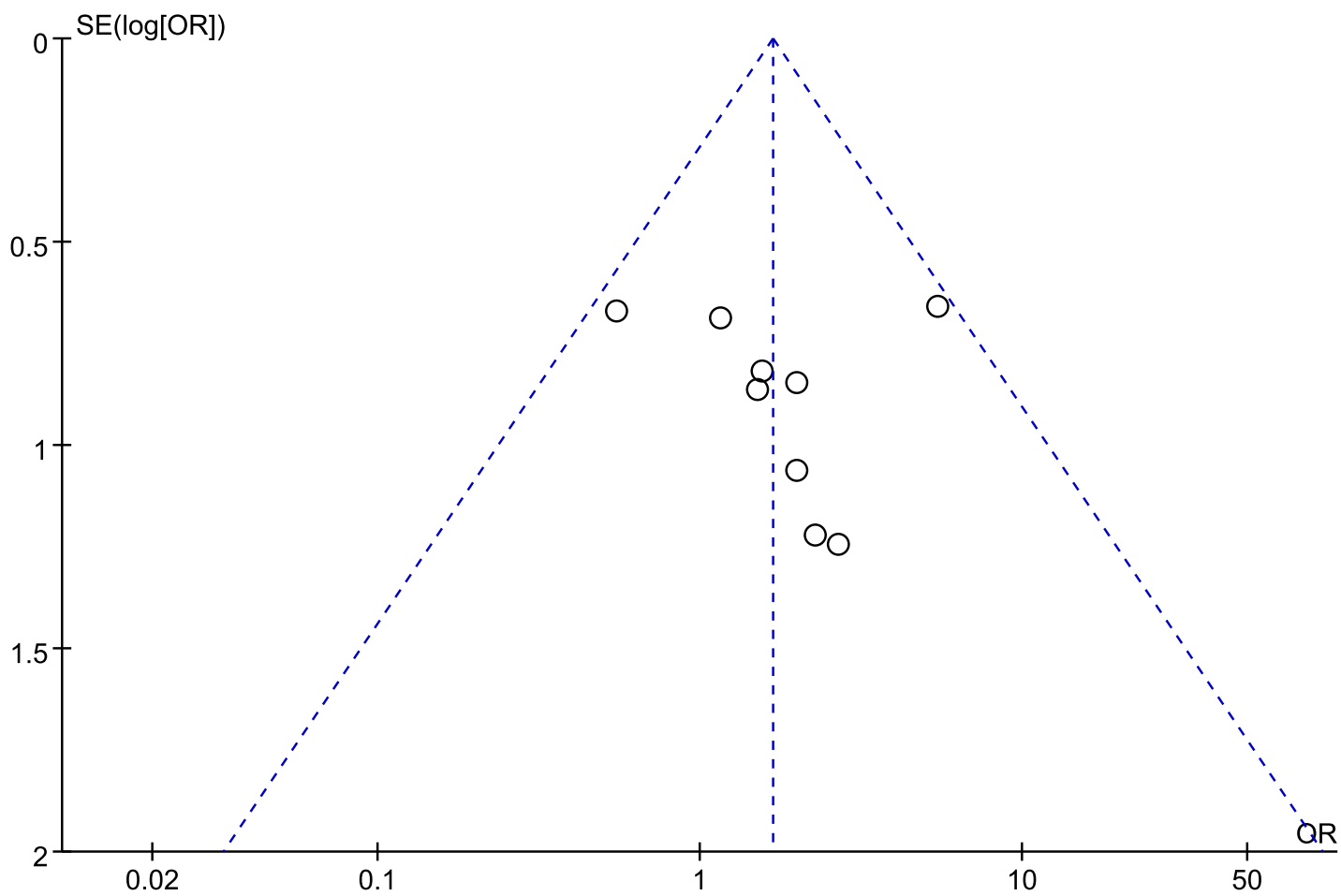

Supplement: Supplementary file 1 [file 2153-8174-26-5-27126-s1.zip › Supplementary Material 2 Funnel plot/Diabetes mellitus Funnel plot.pdf]

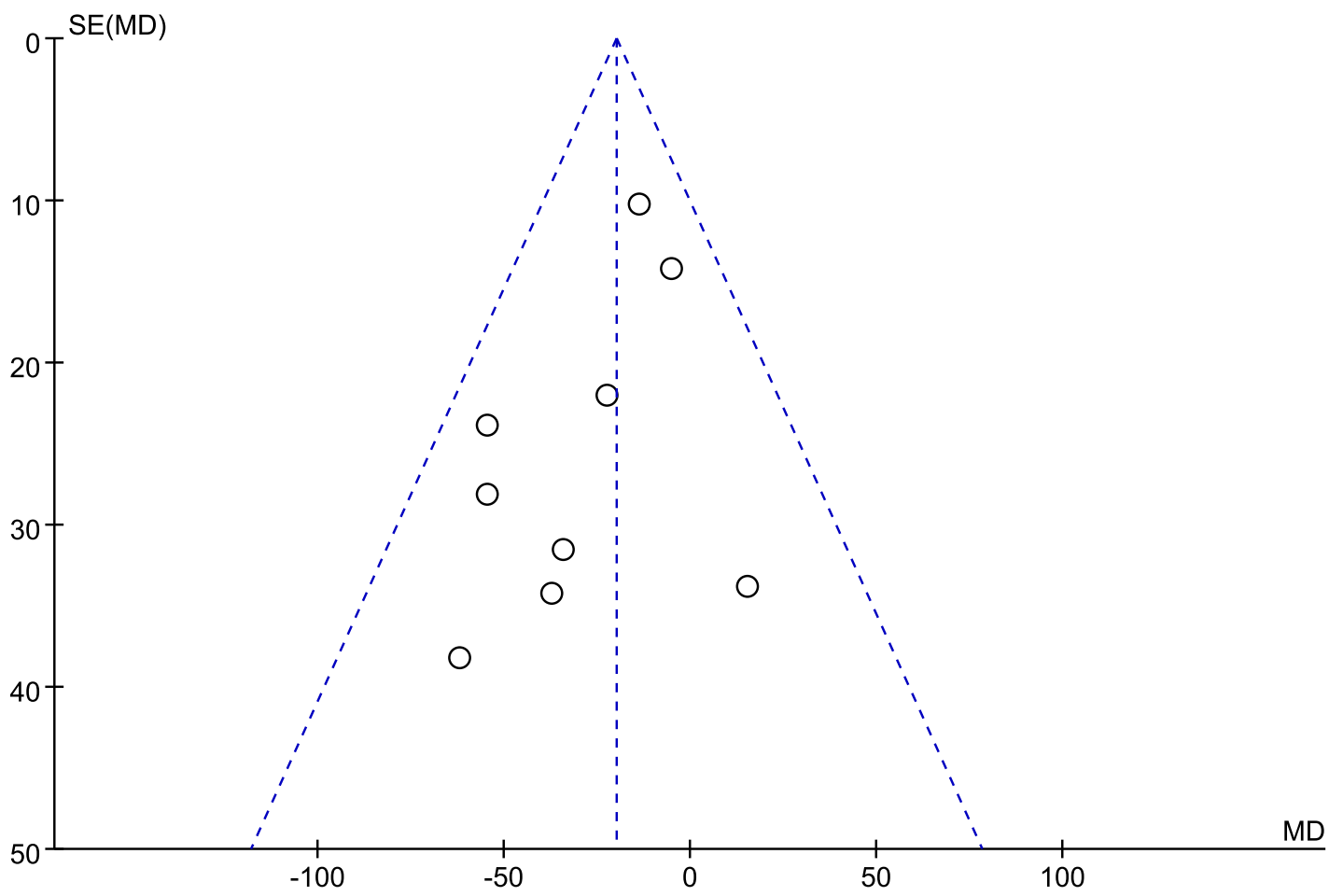

Supplement: Supplementary file 1 [file 2153-8174-26-5-27126-s1.zip › Supplementary Material 2 Funnel plot/ECMO duration Funnel plot.pdf]

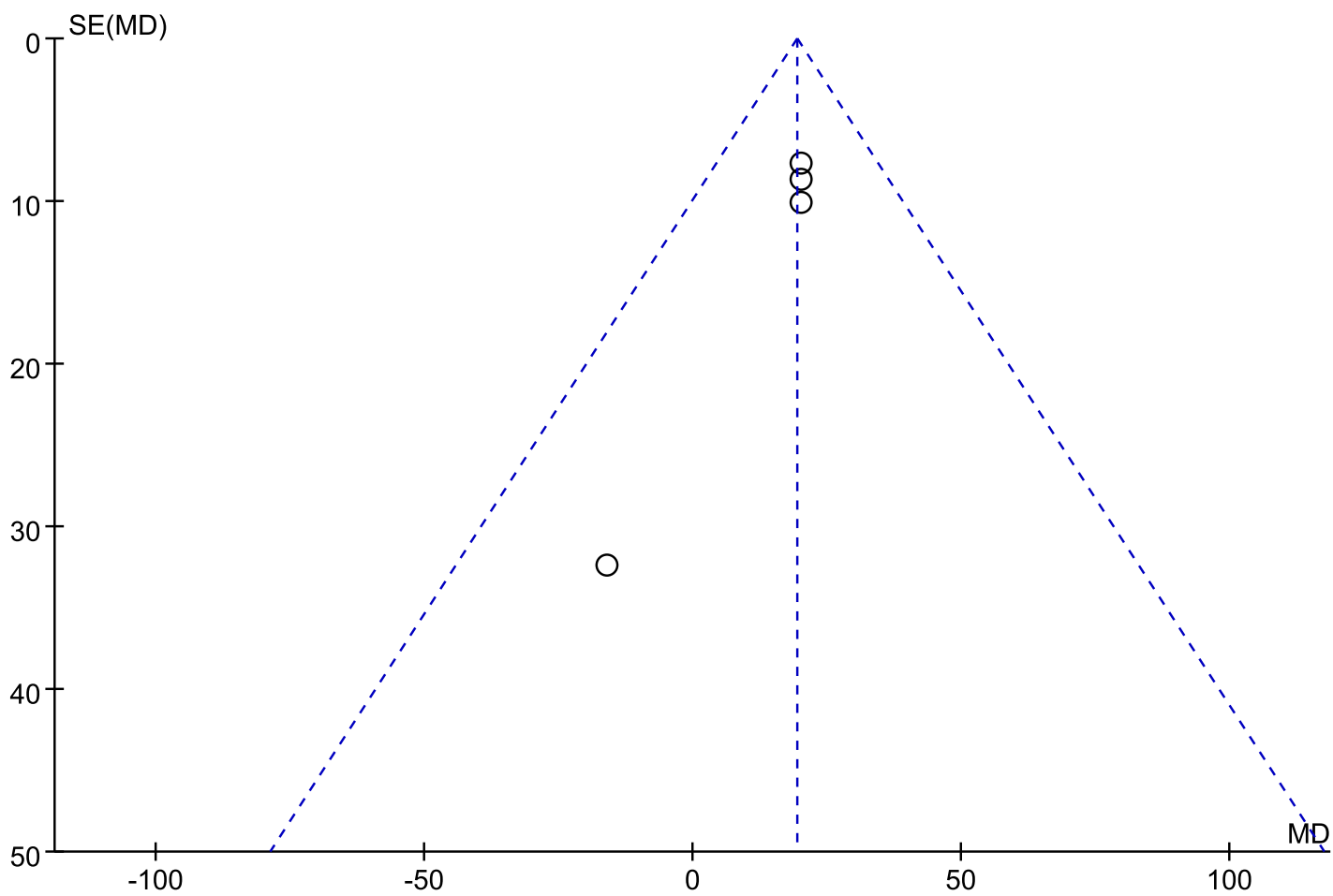

Supplement: Supplementary file 1 [file 2153-8174-26-5-27126-s1.zip › Supplementary Material 2 Funnel plot/heart rate Funnel plot.pdf]

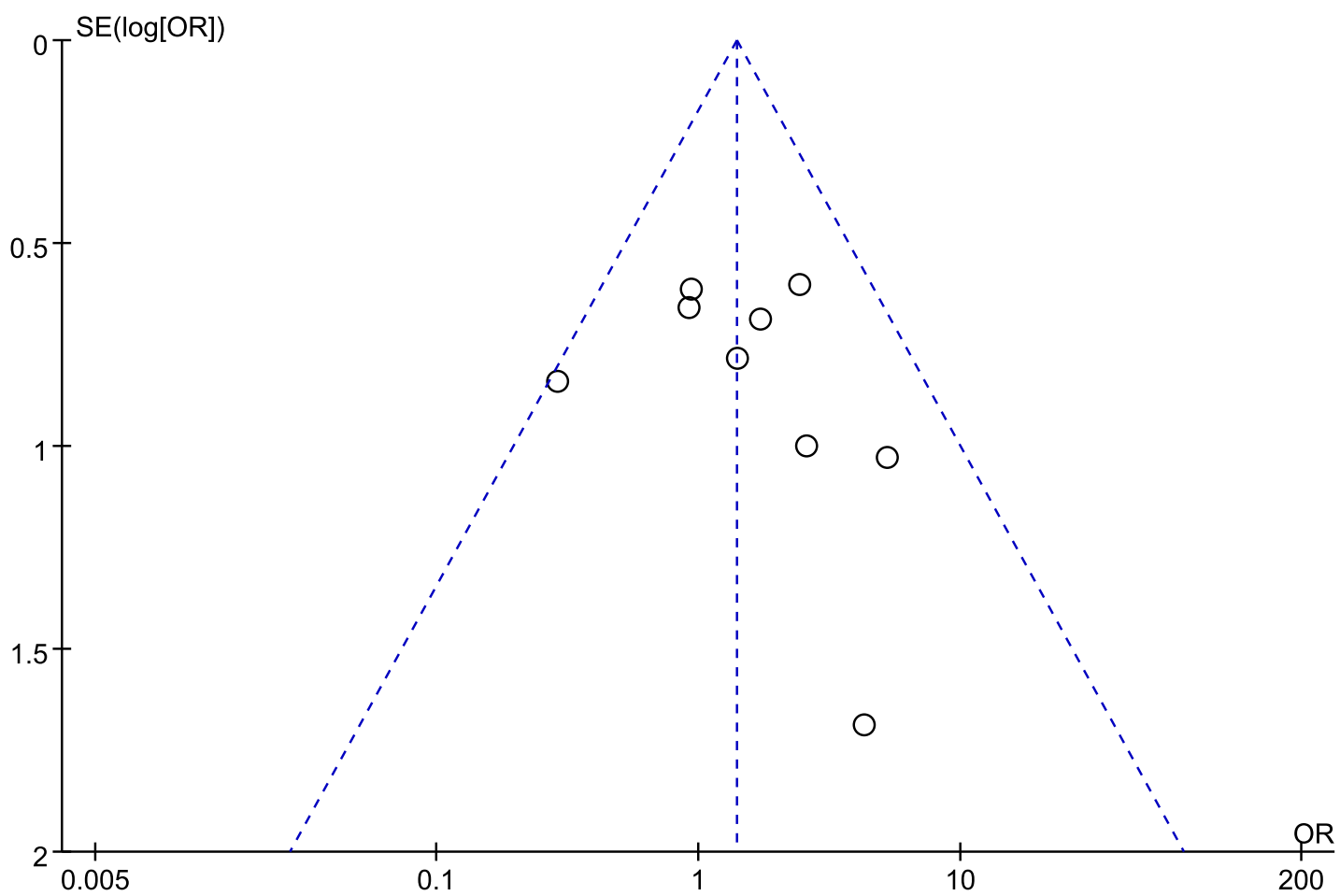

Supplement: Supplementary file 1 [file 2153-8174-26-5-27126-s1.zip › Supplementary Material 2 Funnel plot/hypertension Funnel plot.pdf]

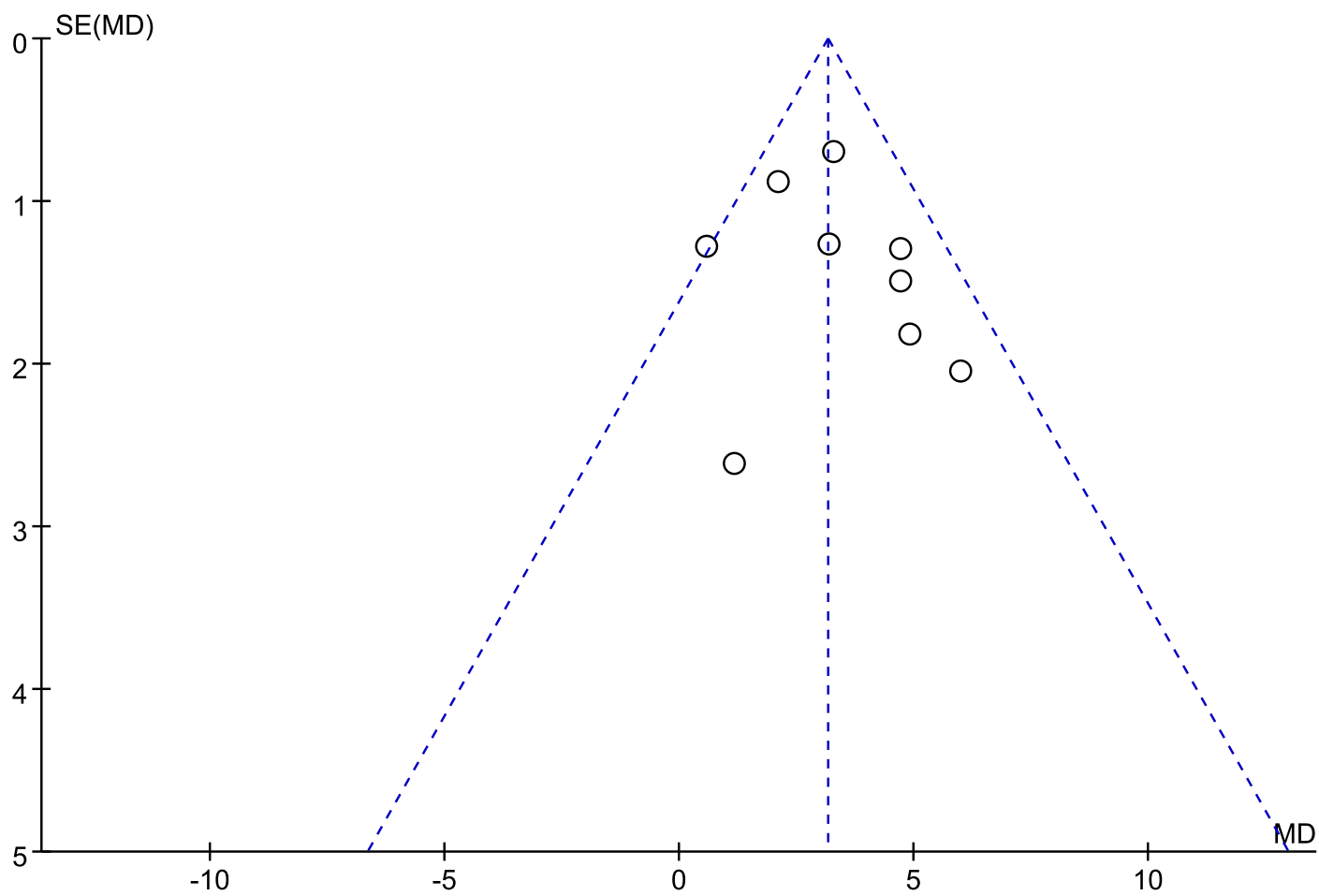

Supplement: Supplementary file 1 [file 2153-8174-26-5-27126-s1.zip › Supplementary Material 2 Funnel plot/Lactate Funnel plot.pdf]

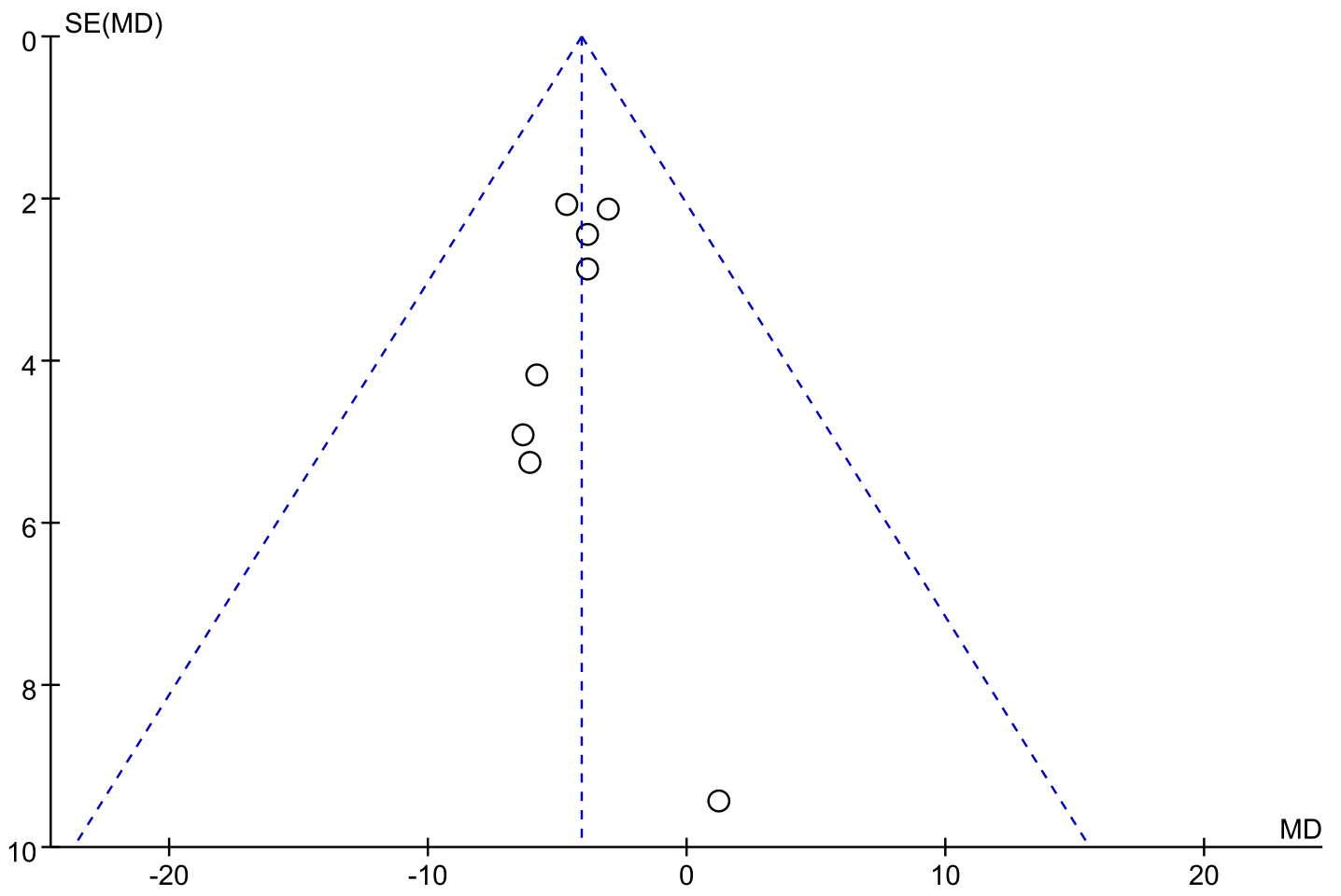

Supplement: Supplementary file 1 [file 2153-8174-26-5-27126-s1.zip › Supplementary Material 2 Funnel plot/LVEF Funnel plot.pdf]

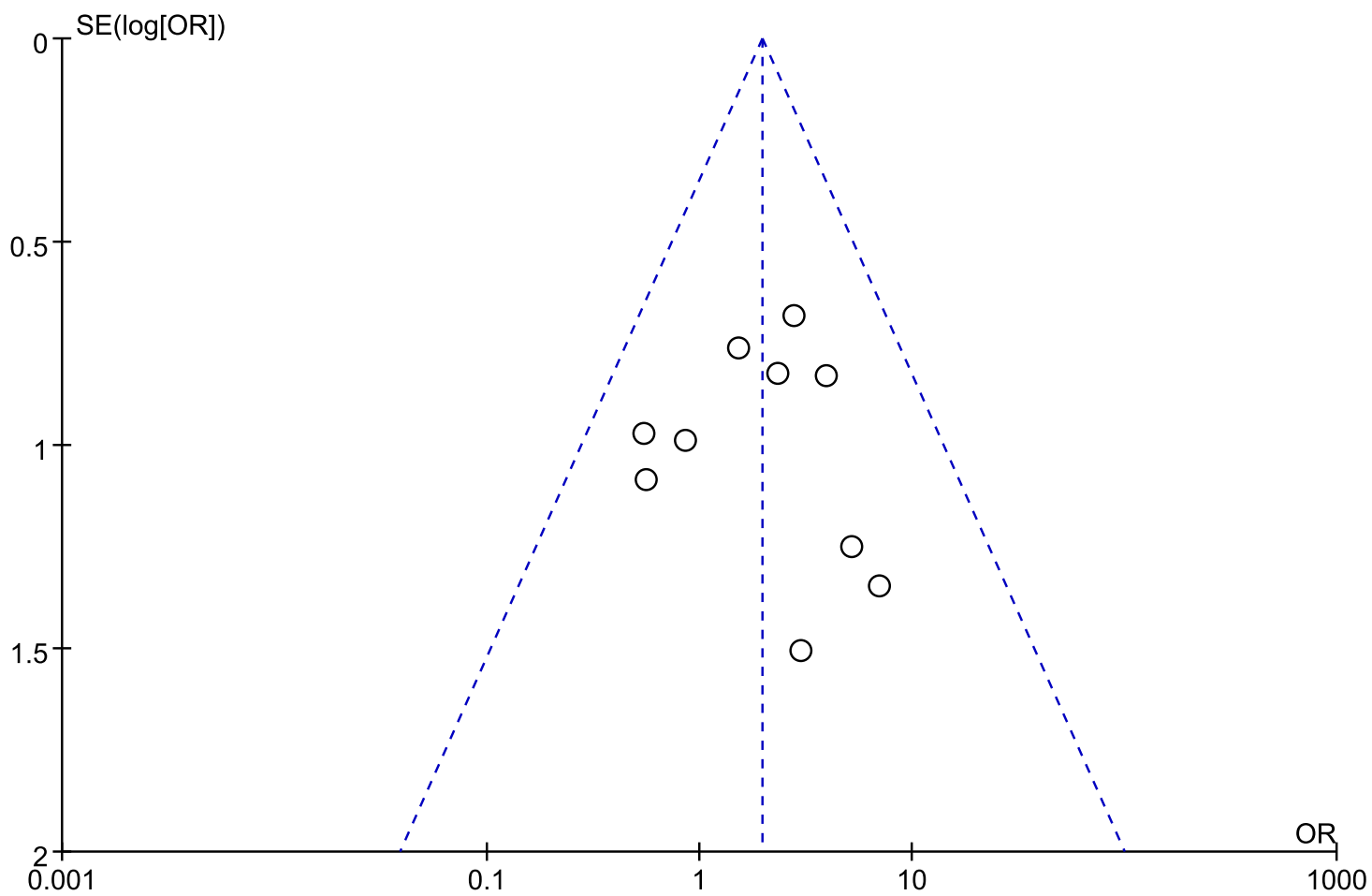

Supplement: Supplementary file 1 [file 2153-8174-26-5-27126-s1.zip › Supplementary Material 2 Funnel plot/Male Funnel plot.pdf]

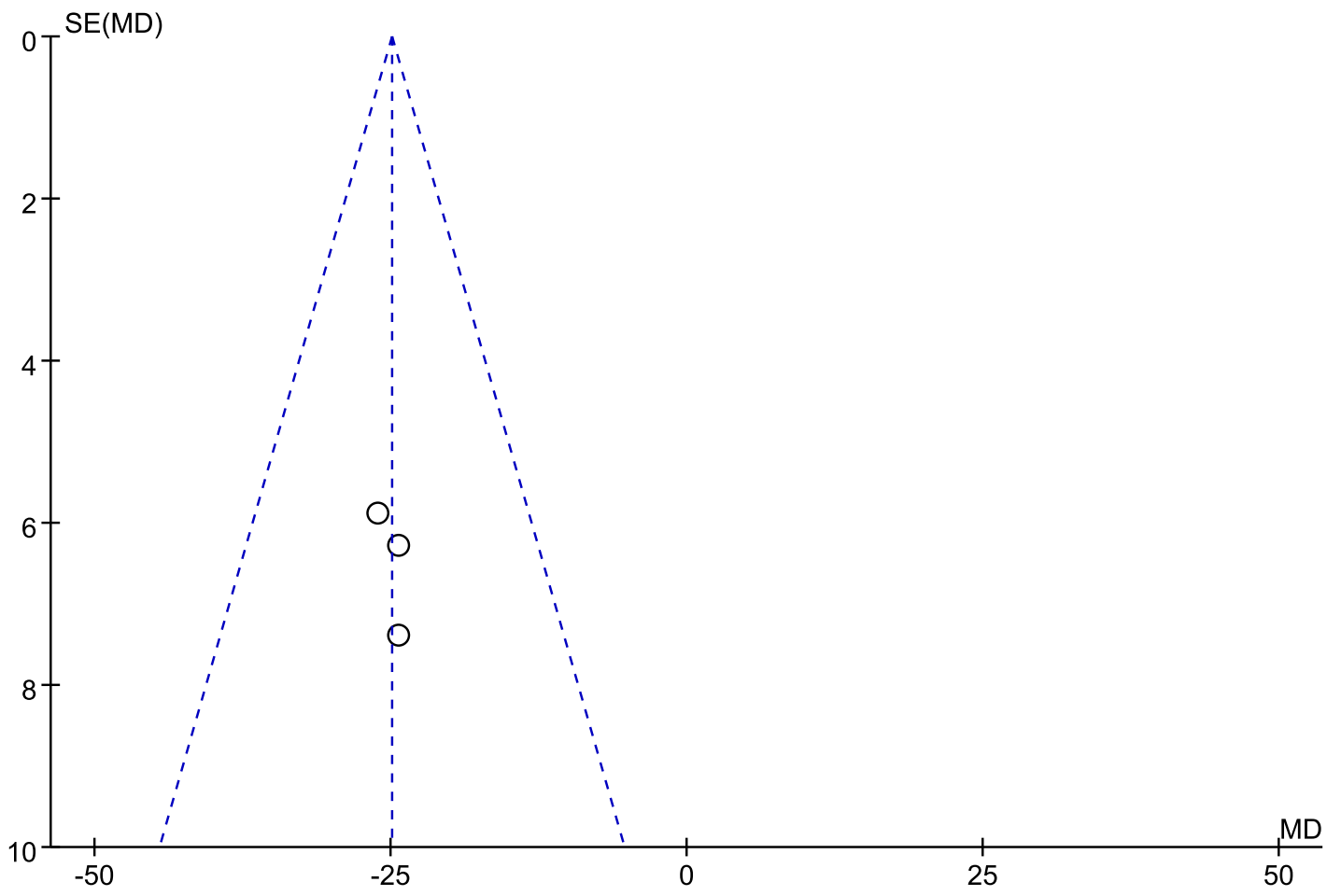

Supplement: Supplementary file 1 [file 2153-8174-26-5-27126-s1.zip › Supplementary Material 2 Funnel plot/MAP Funnel plot.pdf]

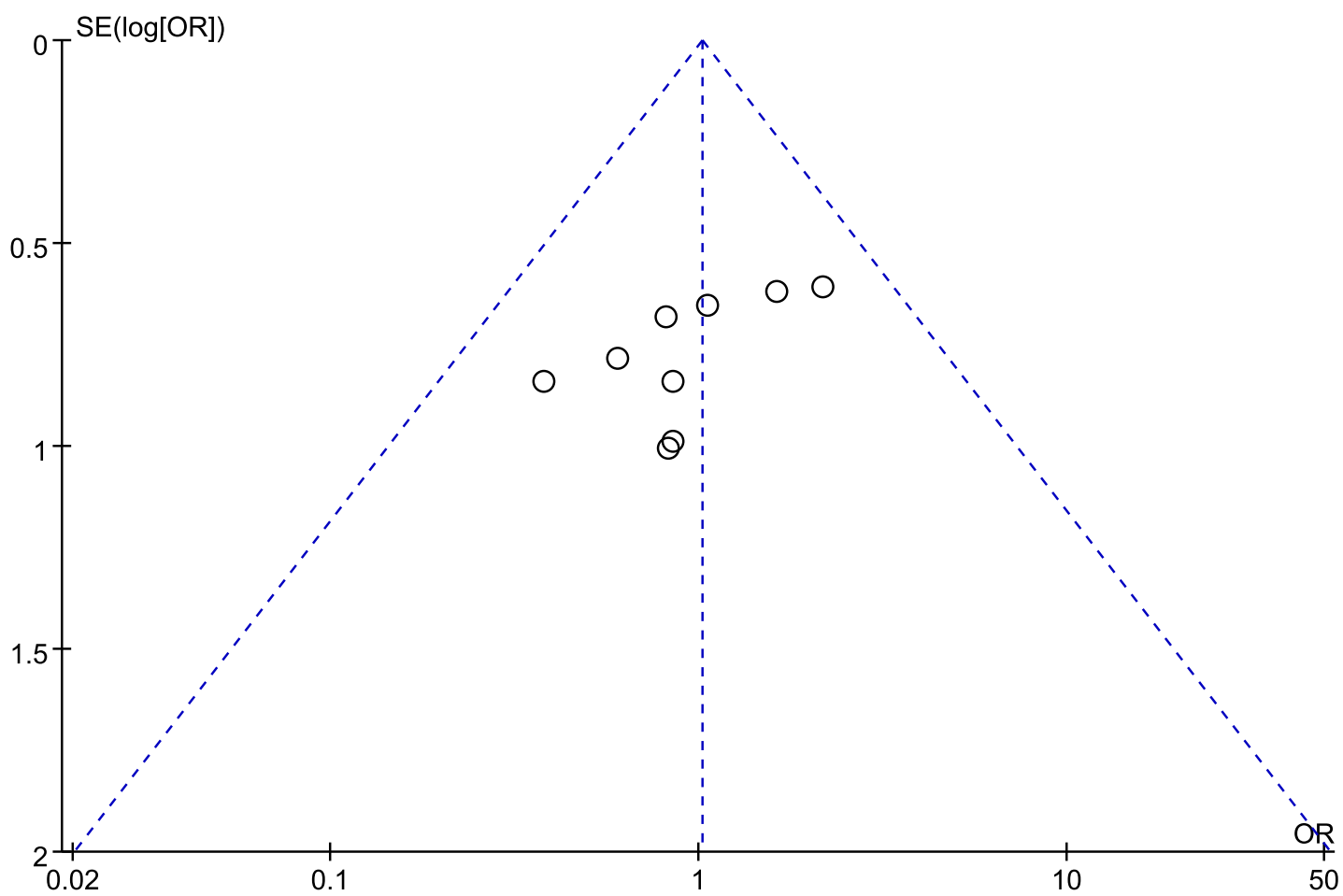

Supplement: Supplementary file 1 [file 2153-8174-26-5-27126-s1.zip › Supplementary Material 2 Funnel plot/Smoking history Funnel plot.pdf]

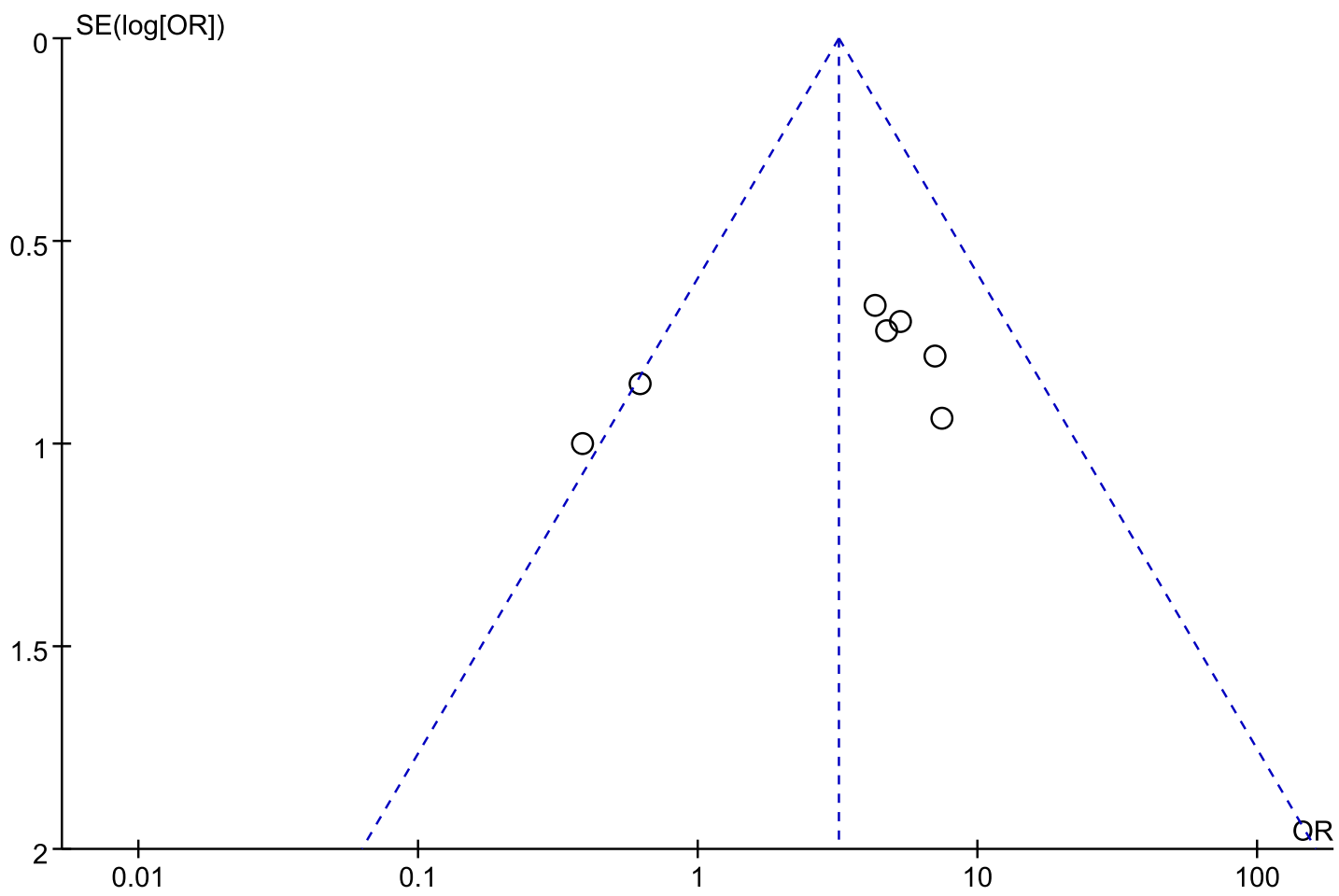

Supplement: Supplementary file 1 [file 2153-8174-26-5-27126-s1.zip › Supplementary Material 2 Funnel plot/Type of infarction-associated CA-Left anterior descending Funnel plot.pdf]

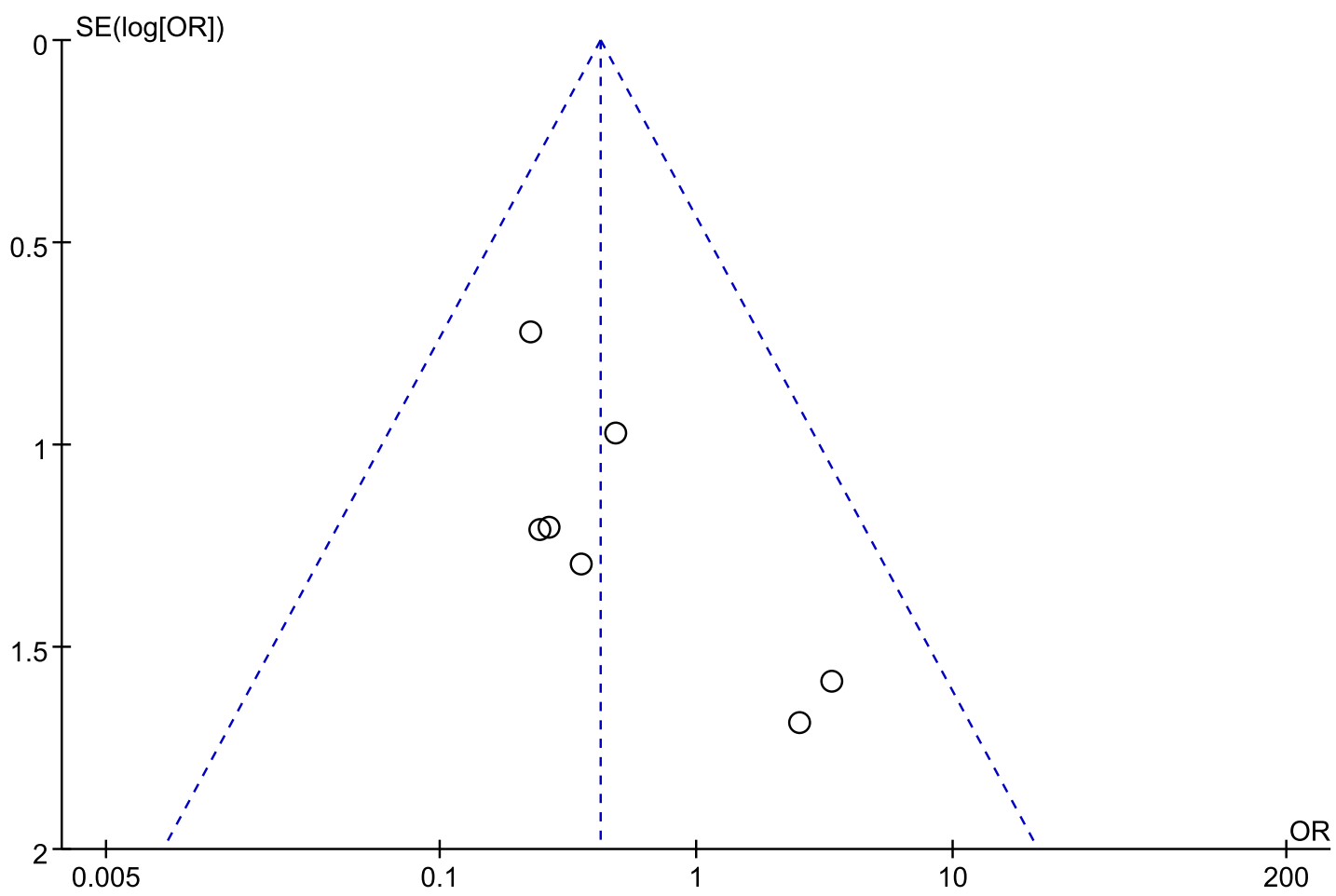

Supplement: Supplementary file 1 [file 2153-8174-26-5-27126-s1.zip › Supplementary Material 2 Funnel plot/Type of infarction-associated CA-Left circumflex CA Funnel plot.pdf]

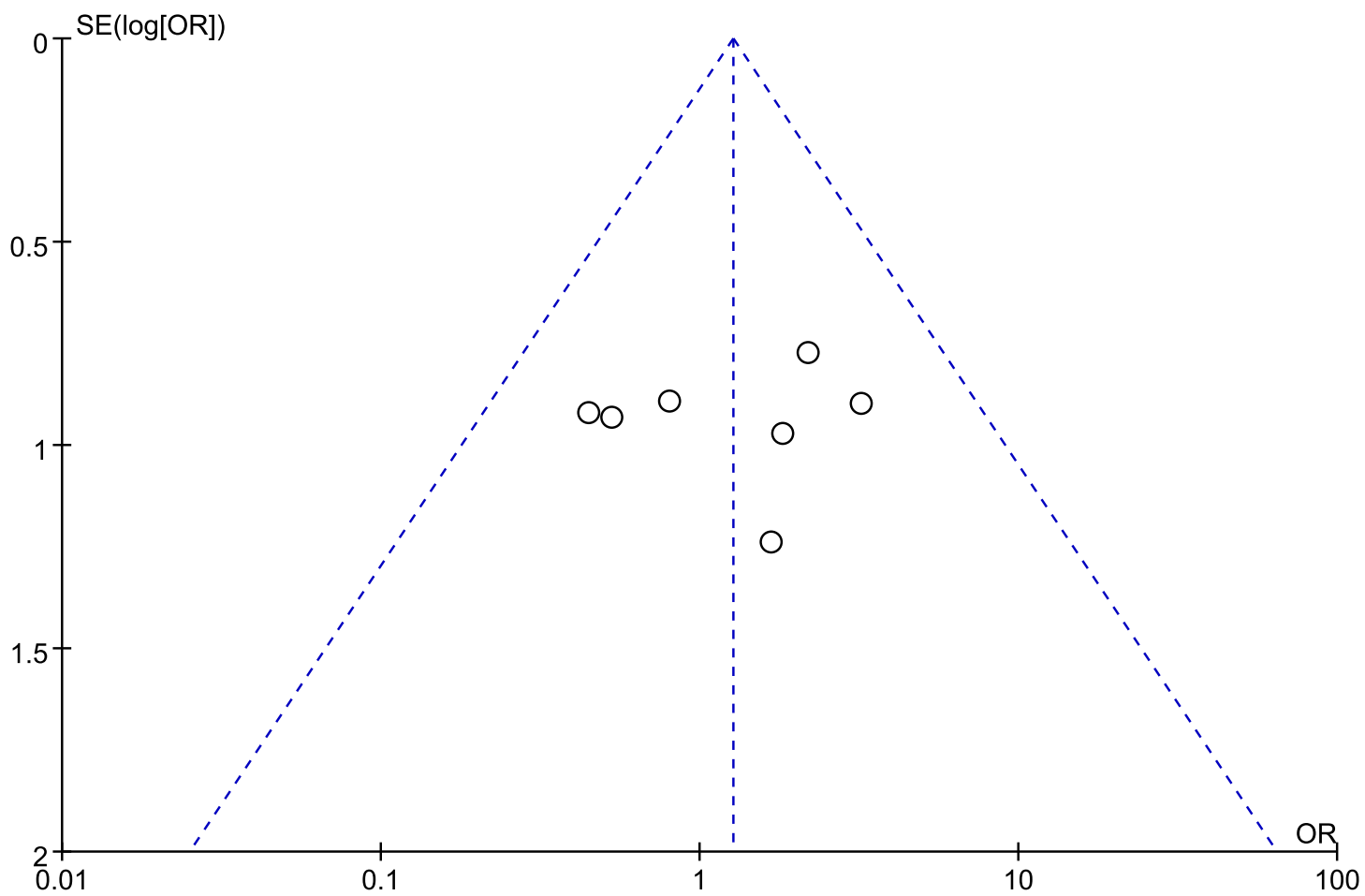

Supplement: Supplementary file 1 [file 2153-8174-26-5-27126-s1.zip › Supplementary Material 2 Funnel plot/Type of infarction-associated CA-Left main CA Funnel plot.pdf]

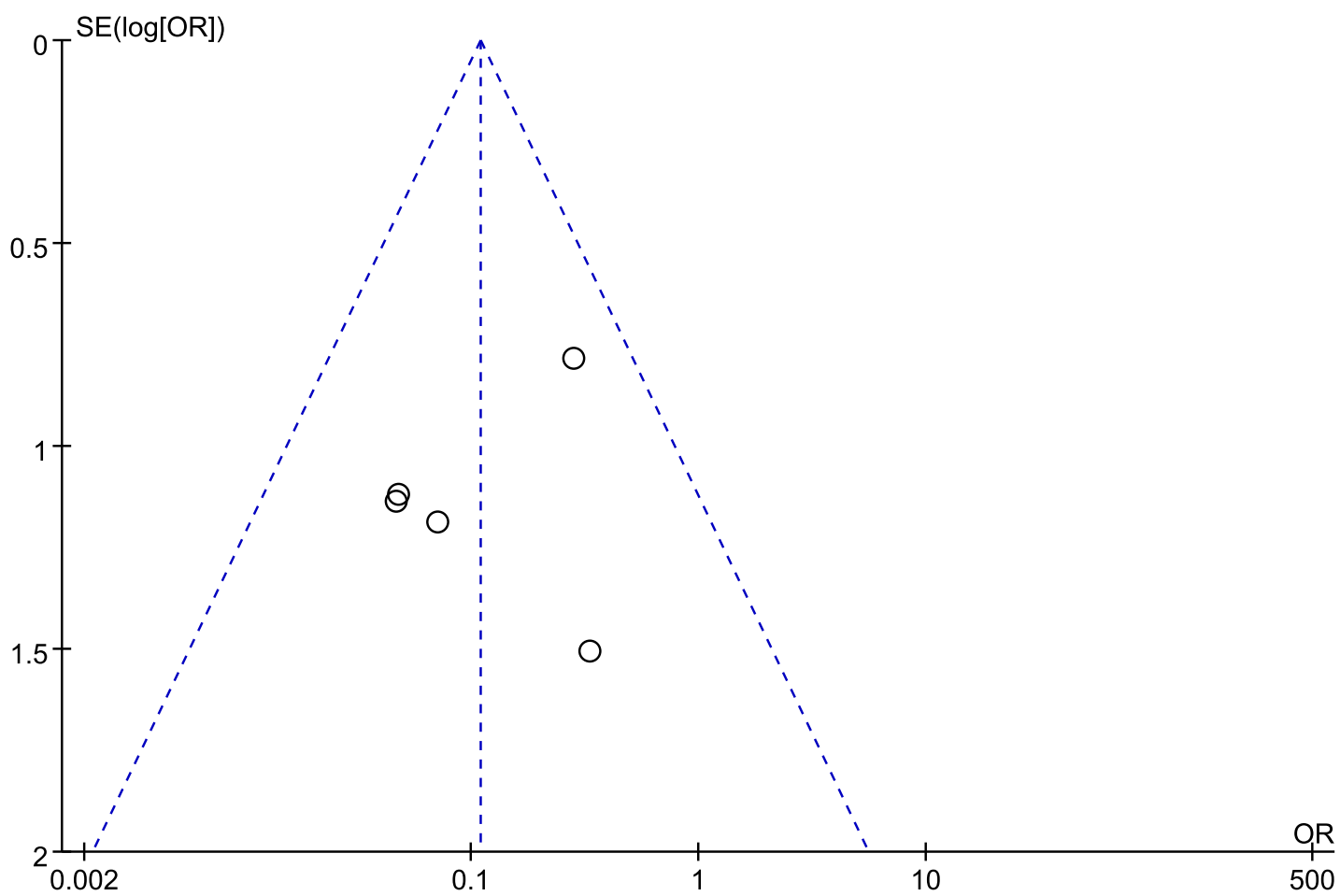

Supplement: Supplementary file 1 [file 2153-8174-26-5-27126-s1.zip › Supplementary Material 2 Funnel plot/Type of infarction-associated CA-RCA Funnel plot.pdf]

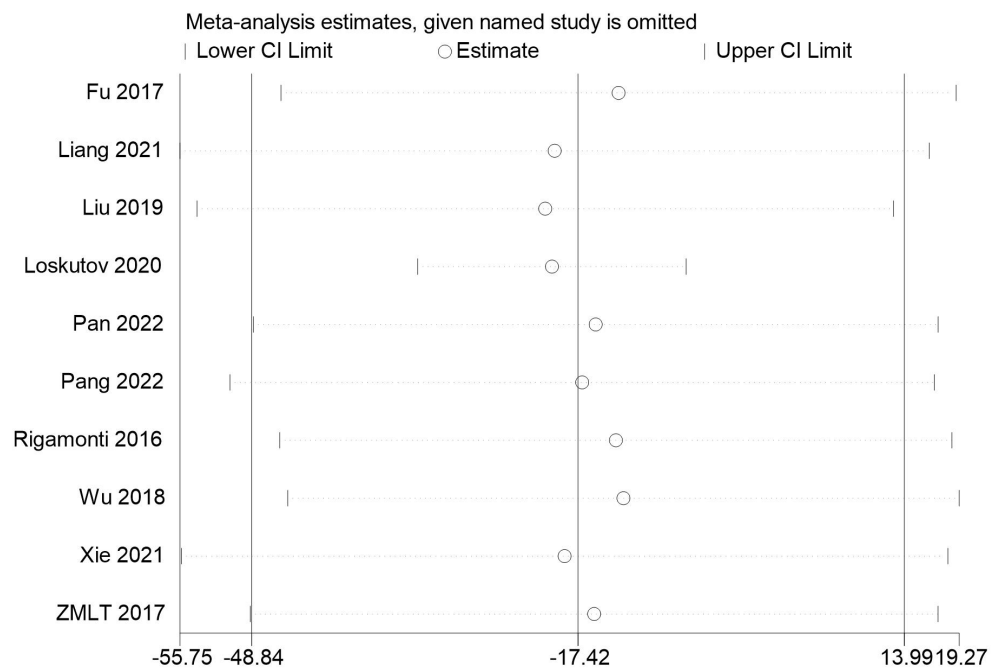

Supplement: Supplementary file 1 [file 2153-8174-26-5-27126-s1.zip › Supplementary Material 4 Sensitivity analysis/ECMO duration.pdf]

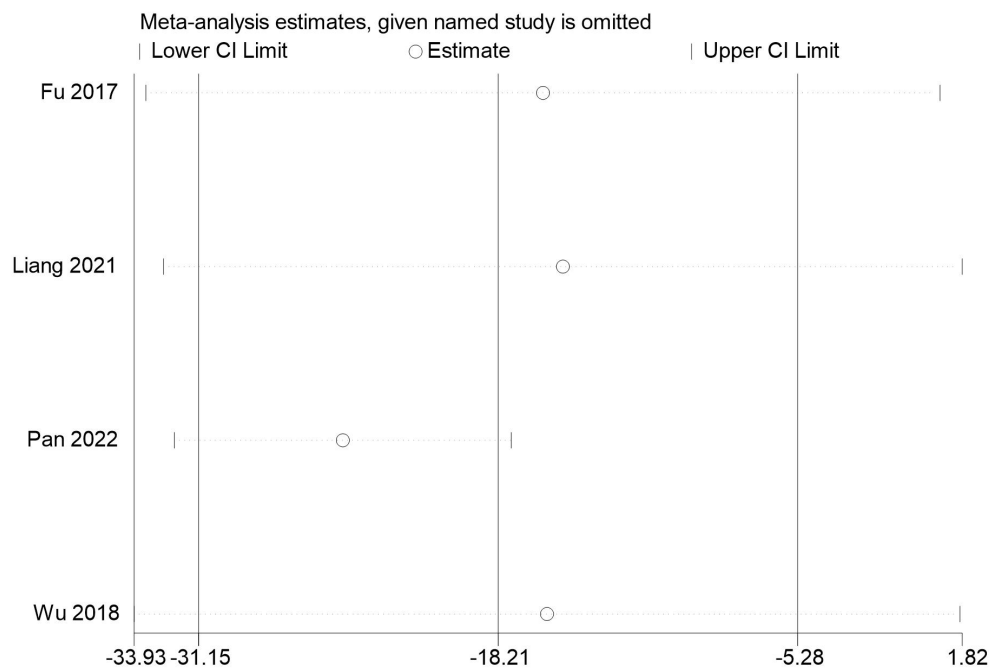

Supplement: Supplementary file 1 [file 2153-8174-26-5-27126-s1.zip › Supplementary Material 4 Sensitivity analysis/MAP.pdf]

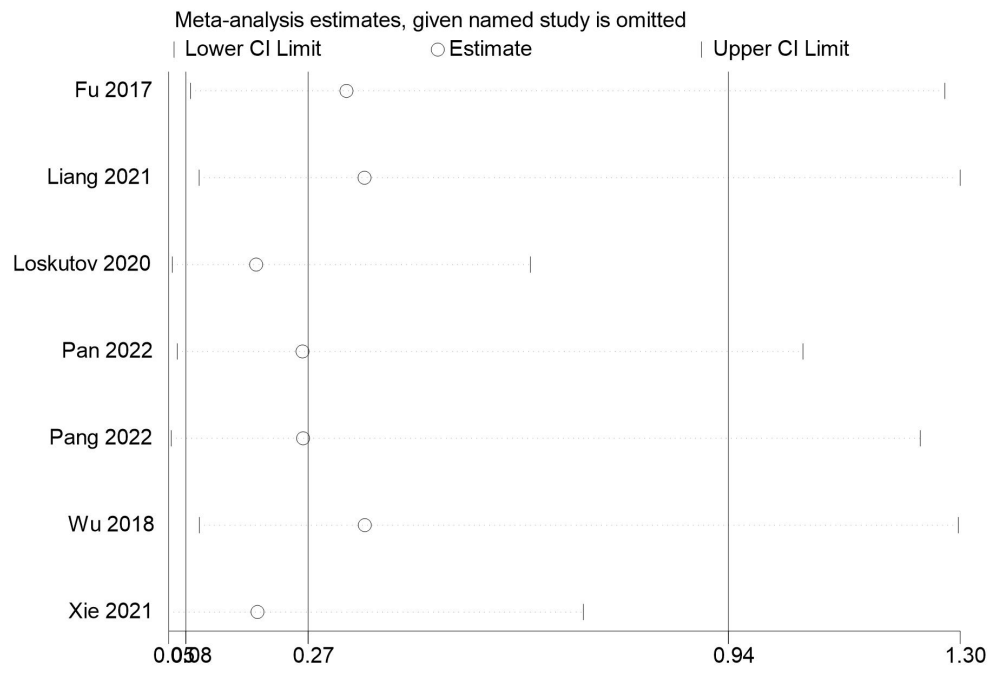

Supplement: Supplementary file 1 [file 2153-8174-26-5-27126-s1.zip › Supplementary Material 4 Sensitivity analysis/Type of infarction-associated CA-RCA.pdf]
